# Ligand-Wise Stripping Dictates Metal Ensemble Catalysts for Selective Oxidation of Biomass-Derived 5-Hydroxymethylfurfural

**DOI:** 10.1007/s40820-026-02118-7

**Published:** 2026-03-23

**Authors:** Junkai Li, Guanhua Wang, Yunxiang Wu, Chuqiao Song, Tairan Pang, Zechao Zhuang, Jiarui Yang, Wenjie Sui, Lili Lin, Dingsheng Wang, Ligang Wang, Chuanling Si

**Affiliations:** 1 State Key Laboratory of Bio-based Fiber Materials, Tianjin Key Laboratory of Pulp and Paper, College of Light Industry Science and Technology, Tianjin University of Science and Technology, Tianjin, 300457 People’s Republic of China; 2 Institute of Industrial Catalysis, State Key Laboratory of Green Chemistry Synthesis Technology, College of Chemical Engineering, Zhejiang University of Technology, Hangzhou, 310014 People’s Republic of China; 3 Department of Chemistry, Tsinghua University, Beijing, 100084 People’s Republic of China; 4 State Key Laboratory of Food Nutrition and Safety, College of Food Science and Engineering, Tianjin University of Science and Technology, Tianjin, 300457 People’s Republic of China; 5 Institute of Molecular Plus, Tianjin University, Tianjin, 300072 People’s Republic of China

**Keywords:** Atom cluster catalysts, Single atom, Cluster synergistic catalysis, Cascade oxidation

## Abstract

Single-atom catalysts (SACs), with their well-defined active sites, demonstrate remarkable selectivity in biomass platform chemical conversions. However, the feature of single active site fails to synergistically regulate the divergent oxidation pathways of multiple functional groups, thereby restricting multi-step reaction efficiency. Herein, a “stepwise N-stripping” strategy is developed via lignin-derived N-doped carbon matrices, which achieves controlled evolution from isolated Co-N_4_ single site to multi-scale atomic/cluster Co synergistic sites by precisely modulating the pyrolysis pathways of lignin-Co precursors. Theoretical and experimental evidence elucidates a synergy-enhanced tandem dual-site catalytic mechanism that single Co atoms with Co-N_2_ configuration selectively activate aldehyde groups to drive sequential carboxylation (5-hydroxymethylfurfural (HMF) to 5-hydroxymethyl-2-furancarboxylic acid (HMFCA) and 5-formyl-2-furoic acid (FFCA) to 2,5-furandicarboxylic acid (FDCA)), while Co clusters enhance oxygen activation for accelerated hydroxymethyl oxidation (HMFCA to FFCA). As expected, the optimized Co-N_2_/Co_4_ dual-site catalyst achieves 98.76% FDCA yield at 55 °C, surpassing most reported supported metal catalysts, alongside robust cycling stability (6 cycles with > 97% FDCA yield). This work establishes a biomass-tailored paradigm for constructing atomic/cluster hybrid catalysts and unravels the dynamic cooperation mechanism between distinct active sites in multi-step oxidation, advancing the rational design of efficient systems for biomass valorization.

**Supplementary Information:**

The online version contains supplementary material available at 10.1007/s40820-026-02118-7.

## Highlights

- Lignin-tailored Co-N_2_/Co_4_ catalyst with tunable active sites were constructed by the “stepwise N-stripping” strategy.
- Co-N_2_ and Co_4_ sites collaboratively drove cascade oxidation of –CH_2_OH and –CHO groups.
- A record 2,5-furandicarboxylic acid yield of 98.76% was achieved under mild conditions over the dual-site catalyst.

**Supplementary Information:**

The online version contains supplementary material available at 10.1007/s40820-026-02118-7.

## Graphical abstract

**Figure:**
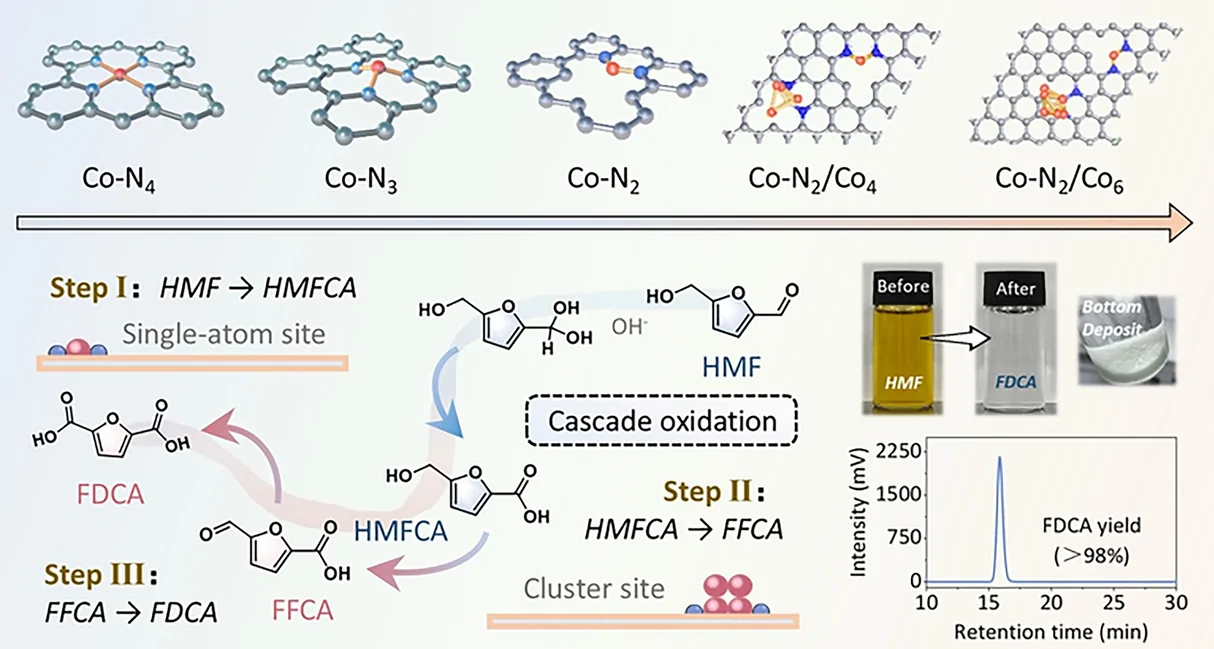

## Introduction

While fossil fuels have underpinned modern industrial civilization through the production of critical chemicals—from polymer feedstocks to petrochemical intermediates, their unsustainable exploitation has precipitated dual crises in energy security and ecological integrity [1–3]. Lignocellulose, as an abundant carbon–neutral feedstock, offers a sustainable alternative for petroleum-derived products. Cellulose-derived C6 sugars (glucose/fructose) undergo acid-catalyzed dehydration to yield 5-hydroxymethylfurfural (HMF), whose rigid furan architecture and bifunctional groups (hydroxymethyl and aldehyde) enable significant potential for industrial applications [4, 5]. This positions HMF as the foremost biorefinery platform molecule, particularly as its catalytic oxidation to 2,5-furandicarboxylic acid (FDCA) via scalable processes represents the most studied pathway [6, 7].

FDCA, derived from the further oxidation of HMF, has emerged as a promising candidate for synthesizing bio-based polyester materials due to its striking structural similarity to petroleum-derived terephthalic acid. Poly (ethylene 2,5-furandicarboxylate), obtained through copolymerization of FDCA with ethylene glycol, exhibits superior gas barrier properties (oxygen and carbon dioxide), thermal stability, and biocompatibility compared to conventional petroleum-based polyethylene terephthalate, positioning it as a viable large-scale alternative in food packaging and engineering plastics [8, 9]. However, the conversion of HMF to FDCA involves a multi-step cascade oxidation process (hydroxymethyl to aldehyde to carboxylic acid), which requires the synergistic interplay of distinct active sites to achieve C-H bond and oxygen radical activation as well as deep oxidation of reaction intermediates [10–12]. Conventional catalysts often exhibit limited control over reaction pathways due to the lack of multifunctional active sites, particularly under mild conditions where balancing high activity and selectivity remains a critical challenge [13, 14].

Single-atom catalysts (SACs), characterized by their atomically dispersed active sites, near-100% atomic utilization efficiency, and tunable coordination structures, offer a novel paradigm to address the aforementioned catalytic challenges [15–18]. However, the homogeneous single-site configuration may lead to monotonous coordination microenvironments of active centers, thereby restricting dynamic modulation of multi-step synergistic catalysis in complex reactions involving cascade oxidation pathways with multiple intermediates [19–21]. The key breakthrough lies in the rational construction of a “single-atom/cluster” dual-site cooperative system through controlled introduction of trace metal clusters into SACs, which represents a viable strategy to address this challenge [22–24]. This innovative architecture enables synchronized modulation of critical kinetic processes by flexibly balancing energy barriers across sequential reaction steps, offering an elegant and efficient strategy for sophisticated oxidation systems [25, 26].

Herein, this study presents a facile thermal treatment strategy for the controlled synthesis of Co single-atom/cluster catalysts with the tailored coordination environment, achieved by modulating the pyrolysis of lignin-metal precursors. Lignin, a key lignocellulosic component and the most abundant aromatic biopolymer, serves as a compelling precursor for SACs, leveraging its phenolic groups to coordinate metal ions and high carbon content to form stable matrices via pyrolysis [27–29]. Combined experimental and density functional theory (DFT) studies reveal that the stepwise nitrogen-stripping cascade reaction mechanism selectively generates low-coordination Co-N_2_ sites with enhanced activity toward aldehyde-to-carboxylic acid oxidation, then the introduction of Co_4_ clusters promotes oxidation of hydroxymethyl side chain C-H bonds. Further mechanistic insights emerge from quantitative analysis of oxygen radical species in single-atom versus dual-active systems, highlighting the critical role of Co_4_ clusters in oxygen activation to accelerate the 5-hydroxymethyl-2-furancarboxylic acid (HMFCA) to 5-formyl-2-furoic acid (FFCA) conversion. The synergistic Co single-atom/cluster system endows the catalyst with excellent selectivity, enabling efficient HMF-to-FDCA conversion with a remarkable FDCA yield of 98.76% under low temperature (55 °C), which represents a leading performance in related research. This work not only advances the rational design of high-performance metal SACs with synergistic atomic/cluster architectures, but also deciphers the fundamental principles underlying dual-site cooperativity in enhancing catalytic efficiency for alcohol/aldehyde-functionalized biomass transformations.

## Experimental Section

### Preparation of Lignin-Tailored Co Single-Atom/Cluster Catalysts

Enzymatic hydrolysis lignin (0.6 g) and NaOH (0.1 g) were dissolved in deionized water to produce a lignin solution with a pH of 12.5. The metal solution was fabricated by dissolving 0.15 mmol CoCl_2_·6H_2_O and 1.35 mmol ZnCl_2_ in 300 mL deionized water. Add hydrochloric acid (10 μL) to the metal solution to minimize the interference of cobalt and zinc hydroxides in the system. Subsequently, the metal solution was gradually dropped into the lignin solution at a rate of 6 mL min^−1^, while magnetic stirring was implemented constantly. After stirring at 500 rpm for 120 min, the Co-Zn/lignin complex was harvested through concentration.

The Co-Zn/lignin precursor was obtained by mixing the Co-Zn/lignin complex with 9.0 g melamine through ultrasonic-assisted stirring and then freeze-drying. Subsequently, the Co-Zn/lignin precursor underwent programmed pyrolysis in a tube furnace under N_2_ atmosphere via the thermal sequence: 5 °C min^−1^ ramp to 550 °C (1 h hold), identical ramp to 900 °C (1 h hold), followed by direct heating to 1050 °C with respective 1 and 2 min isothermal holds, affording Co-N_2_/Co_4_ and Co-N_2_/Co_6_ SACs. For comparative purposes, we also prepared Zn-loaded (Co-free) and metal-free N-doped lignin-derived carbon-based catalysts (NC), and the detailed preparation protocol has been presented in the Supporting Information. The detailed characterization protocols of the catalyst samples are provided in the Supporting Information.

### Preparation of Lignin-Tailored Co Single-Atom Catalysts

The Co-Zn/lignin precursor was heated in a tube furnace under a N_2_ atmosphere at 550 and 900 °C, respectively, for 1 h with a heating rate of 5 °C min^−1^. After pyrolysis, the pyrolysis products were immersed in 2 M HCl at 75 °C for 12 h and then rinsed with deionized water to obtain lignin-based Co-N_4_ SACs samples. The preparation process of Co-N_3_ SACs sample is identical to that of Co-N_4_ SACs, but the pyrolysis process was modified slightly. After the pyrolysis protocol similar to Co-N_4_ (at 550 and 900 °C, respectively, for 1 h), the temperature was further elevated from 900 to 1000 °C with the same heating rate and was then kept for 1 min. The Co-N_2_ SACs catalyst was obtained by acid leaching of the Co‑N_2_/Co_4_ sample with 2 M HCl at 75 °C for 12 h.

### Oxidize HMF to FDCA by Co Catalysts

The catalytic oxidation of HMF was conducted in a 50-mL stainless steel reactor. After the reaction, the solid catalyst and liquid components were separated by centrifugation. Regarding the liquid components, the contents of HMF and its oxidation products were determined by HPLC using an Aminex® HPX-87H column with 5 mM sulfuric acid solution as the mobile phase. The solid catalyst was washed with deionized water and dried for the next catalytic test. After the fifth recycling experiment was completed, the separated catalyst sample was regenerated under a hydrogen atmosphere (300 °C for 2 h) for the next cycle test.

## Results and Discussion

### Preparation and Characterization of Lignin-Based Co Catalysts

Lignin is abundant in functional groups, particularly phenolic hydroxyl and carboxylic groups, which possess the capability to bind to metal ions to generate water-insoluble lignin-metal compounds [30, 31]. The introduction of nitrogen sources in the Co/Zn-lignin precursor facilitates the establishment of the fluffy structure and the formation of the Co–N configurations within the SACs [32]. The Zn species in the precursor, serving as a pore-forming agent, volatilizes during the pyrolysis process, providing rich anchoring sites for the target metal Co. The residual trace amount of Zn exerts negligible influence on the catalytic reaction.

**Fig. 1:**
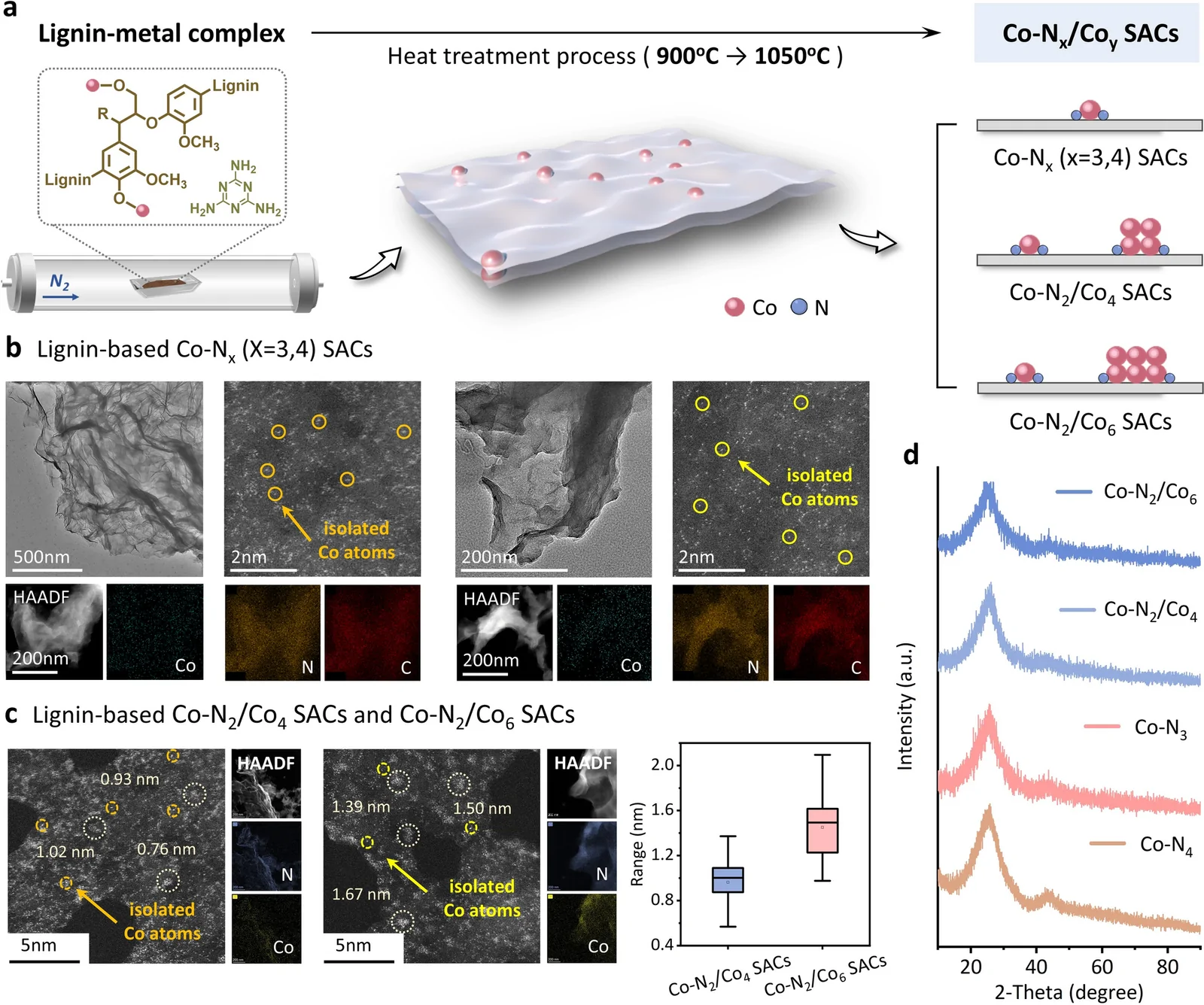
Synthesis and characterization of lignin-based Co catalysts. **a** Synthesis pathway for the preparation of Co single-atom/cluster catalysts; **b** TEM, HAADF-STEM and the EDS mappings of Co-N_3_ (left) and Co-N_4_ SACs (right); **c** HAADF-STEM and the EDS mappings of Co-N_2_/Co_4_ (left) and Co-N_2_/Co_6_ SACs (right), as well as cluster particle size distribution maps of the two catalysts; **d** XRD spectra of Co catalysts

The coordination evolution of Co SACs was systematically modulated through controlled pyrolysis temperature programming. This thermal treatment strategy leverages the distinct thermal stability of nitrogen dopants in the catalyst precursor to achieve progressive removal of coordinated nitrogen atoms from the metal center. At lower pyrolysis temperatures (900 °C), the nitrogen-rich microenvironment facilitates the stabilization of thermodynamically favorable Co-N_4_ structure [33]. With increasing pyrolysis temperature, the graphitization degree of the precursor carbon skeleton is enhanced, and the nitrogen dopant concentration decreases, leading to the removal of partial anchoring sites in Co-N_4_ and ultimately yielding low-coordination Co SACs with distinct catalytic behaviors [34]. Concurrently, the progressive pyrolysis process induces gradual weakening of the bonding energy in Co–N coordination structures under elevated temperatures, which consequently fails to anchor all Co atoms. This releases thermodynamically activated Co species that simultaneously aggregate into clusters with varying particle sizes [35]. Therefore, this thermal activation drives Co–N structural reorganization through continuous bond breaking and reconfiguration, thereby forming lignin-based Co catalysts with single-atom/cluster sites (Fig. 1a).

First, as depicted in the TEM images (Fig. 1b), the prepared Co-N_3_ and Co-N_4_ SACs exhibits an excellent dispersive layered structure with no visible metal spots, suggesting that the Co species are evenly distributed on the support. HAADF-STEM images confirm the atomic dispersion of Co species within the layered architecture, with isolated single Co atoms anchored uniformly across the nitrogen-doped lignin-derived carbon matrix. Elevating the pyrolysis temperature to 1050 °C generates Co clusters (0.97 nm average diameter) alongside isolated Co single-atoms within the Co-N_2_/Co_4_ SACs. Further increasing the thermal treatment intensity induces coalescence of these clusters, resulting in a visible increase in their average diameter to 1.46 nm. The EDX elemental mapping further revealed substantial overlaps between the distribution patterns of Co species and those of C/N in the Co catalysts featuring four distinct coordination environments, thereby implying the presence of chemical interactions between Co and the C/N species (Fig. 1b, c). Notably, the absence of the Co metal nanoparticles diffraction peak in the XRD pattern (Fig. 1d) offered powerful evidence for the synthesis of Co single-atoms or clusters species on the support, which is consistent with the TEM and HAADF-STEM analysis results.

**Fig. 2:**
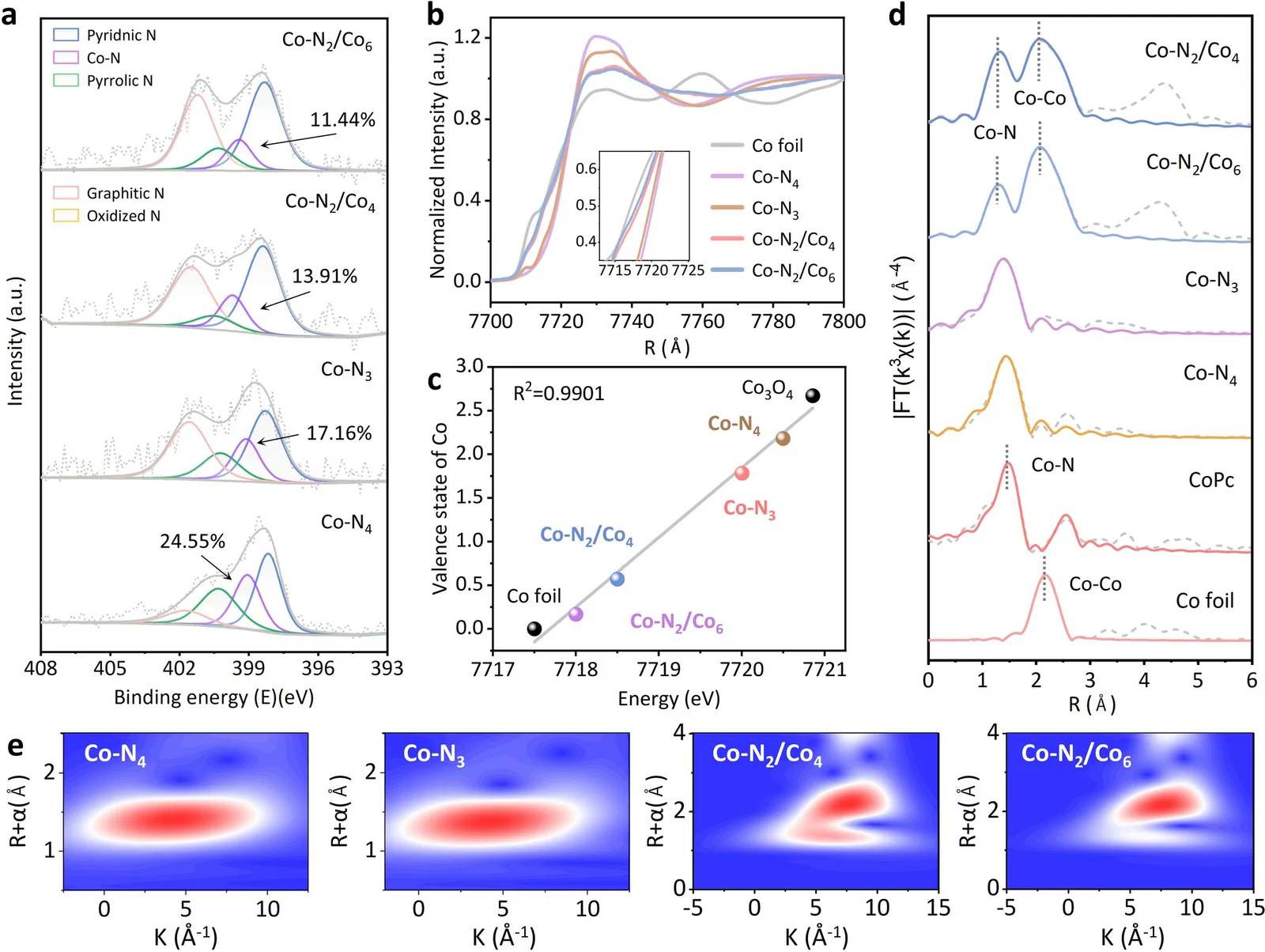
Fine structural characterizations of XPS and XAFS. **a** N 1*s* XPS spectra of Co catalysts; **b, d** XANES and EXAFS spectra of the Co catalysts; **c** Oxidation state of Co species obtained from Co K-edge XANES; **e** WT-EXAFS of Co catalysts

The near-surface elemental composition and valence state of the catalyst samples was analyzed by XPS. According to the XPS spectra, Co single-atom/cluster catalysts are comprised of Co, N, C, and O elements, and the N species in the catalysts are mainly constituted by pyridine nitrogen, Co–N, pyrrole nitrogen and graphite nitrogen (Figs. 2a, S1, and S2). A small shift occurred in the position of pyridine nitrogen in the Co catalysts samples in comparison with the NC catalysts (without Co and Zn salts), which is associated with the Co–N configuration of the Co species and pyridine N coordination, as pyridine nitrogen is generally regarded as a coordination site for Co species (Fig. S3) [36]. Moreover, as pyrolysis severity increases, the abundance of Co–N structure decreases, reflecting the progressive cleavage of partial Co–N bonds within the structure, thereby releasing highly active Co species. Concurrently, ICP-OES analysis revealed that despite the same amount of Co metal added to the catalyst precursors, divergent pyrolysis conditions produced distinct trends in the final Co content (Table S1). The intensified pyrolysis process enhanced Co loading, primarily due to accelerated depletion of N-dopants under more severe thermal treatment, which elevated the proportion of retained Co despite equivalent precursor loading.

Additionally, the increase in the graphitic nitrogen content in Co-N_2_/Co_x_ (x = 4, 6) and Co-N_3_ compared with that in Co-N_4_ SACs might be attributed to the fact that the increase in pyrolysis temperatures promotes the generation of more thermally stable graphitic nitrogen species, thereby reducing the anchoring and dispersing capabilities for the target metal. Furthermore, as evidenced by XPS results (Table S2), the surface concentration ratio of nitrogen in Co–N configurations to Co atoms progressively decreases with increasing pyrolysis temperature, which correlates with the conversion of pyridinic nitrogen to graphitic nitrogen and simultaneously suggests the formation of low-coordination Co–N structures in the catalysts.

Co K-edge X-ray absorption near-edge structure (XANES) spectral analysis reveals that the absorption energy of Co species resides between those of Co foil and Co_3_O_4_ references (Figs. 2b and S4). Quantitative linear combination fitting of XANES profiles demonstrates a progressive reduction in Co oxidation state approaching metallic Co⁰ with increasing pyrolysis temperature or time, indicating the formation of Co⁰ cluster further lowers the average valence state of Co species (Fig. 2c). The coordination environment of Co atoms in Co-N_4_, Co-N_3_, Co-N_2_/Co_4_, and Co-N_2_/Co_6_ was furthermore analyzed by the Fourier-transformed extended X-ray absorption fine structure (EXAFS) (Fig. 2d). The peak at≈1.39 Å is attributed to Co–N coordination, exhibiting coordination numbers of 3.8 (Co-N_4_), 3.2 (Co-N_3_), 2.4 (Co-N_2_/Co_4_), and 1.7 (Co-N_2_/Co_6_), whereas the feature peak of 2.13 Å was corresponded to Co–Co bonding with coordination numbers of 4.1 and 6.0 for Co-N_2_/Co_4_ and Co-N_2_/Co_6_, respectively, further confirming the presence of Co nanoclusters in these systems (Figs. S5 and S6; Tables S3 and S4). In addition, wavelet transform (WT) analysis was employed as a complementary analytical approach to reveal the structural configuration of Co atomic coordination structure. As evidenced in Figs. 2e and S7, the Co-N_2_/Co_4_ and Co-N_2_/Co_6_ SACs exhibit dual intensity maxima in WT profiles compared to Co-N_3_ and Co-N_4_ SACs, attributable to distinct scattering paths of Co–N coordination and emerging Co–Co metallic bonding-finding consistent with the preceding XANES valence state evolution. The diminished Co–N coordination intensity in Co-N_2_/Co_6_ SACs arises from the progressive depletion of Co–N bonds under pyrolysis-driven thermal activation.

### HMF Oxidation Process and Active Site Exploration

**Fig. 3:**
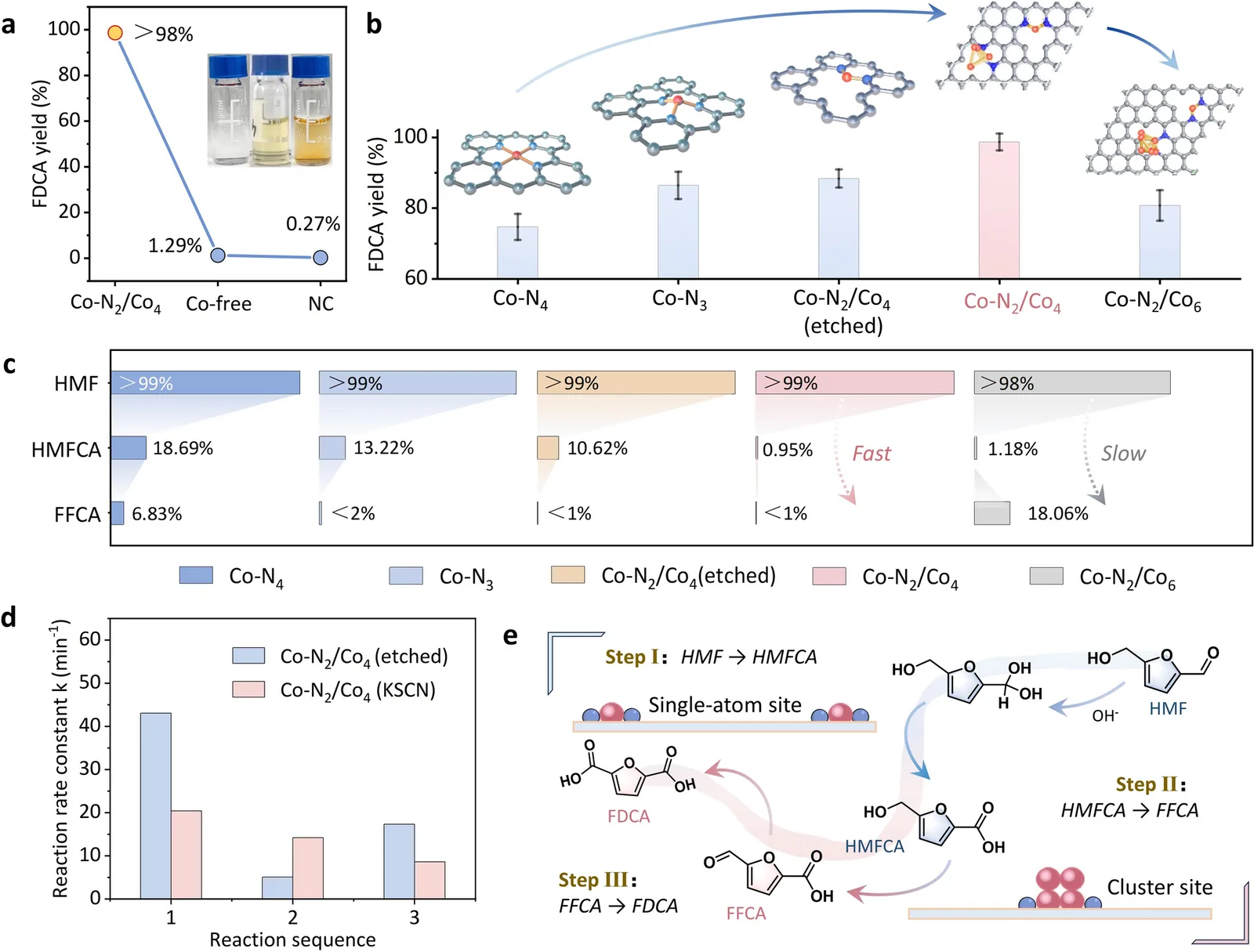
Validating the stepwise enhancement mechanism of dual active sites. **a** Catalytic oxidation of HMF to FDCA over the Co-N_2_/Co_4_ SACs and Zn catalysts (Co-free) and NC catalysts (Metal-free). The illustration presents the actual effect diagrams of the three catalysts; **b**, **c** Investigate the yield variations of FDCA and its intermediates among Co-N_4_, Co-N_3_, Co-N_2_/Co_4_, and Co-N_2_/Co_6_ SACs; **d** Reaction rates of the three catalysts. Reaction sequences 1, 2, and 3, respectively, represent reactions starting from HMF, HMFCA and FFCA; **e** The stepwise oxidation mechanism of single-atom and cluster sites in the HMF oxidation process

The conversion of HMF to FDCA, a pivotal reaction for synthesizing bio-based polyester monomers, represents a critical step in advancing sustainable polymer chemistry. To address the demand for efficient catalysis in this oxidation process, we designed Co single-atom/cluster catalysts supported on lignin-based carbon with distinct metal coordination environments, systematically investigating the mechanisms underlying their enhanced catalytic performance in the oxidation of HMF to FDCA. Insufficient pyrolysis of the catalyst precursor inevitably led to trace Zn species remaining in the catalyst (Fig. S8). Therefore, the effect of non-Co components on the reaction was first verified by comparing the Zn-only catalyst (Co-free) and the metal-free catalyst. Experimental results showed that the catalytic reaction barely occurred under both Co-free (1.29% FDCA yield) and metal-free (0.27% FDCA yield) conditions. Additionally, the yellow supernatant in the reaction system could be attributed to the poor thermal stability of HMF, which underwent thermal polycondensation to form yellow–brown byproducts in the presence of inactive catalysts (Figs. 3a and S9) [32, 37].

Notably, among the four configurations of Co catalysts, the dual-site Co-N_2_/Co_4_ catalyst exhibited the optimal catalytic performance (98.76% FDCA yield) under the same low-temperature conditions (55 °C, 5 bar O_2_) (Fig. 3b). To investigate the enhancement mechanism of the Co-N_2_/Co_4_ dual sites, the Co_4_ sites in the Co-N_2_/Co_4_ catalyst were removed via acid leaching, theoretically yielding a Co-N_2_ configuration catalyst. Although the Co-N_2_/Co_4_ (etched) catalyst still showed the best catalytic performance among Co SACs (Co-N_3_ and Co-N_4_ SACs), its FDCA yield decreased by approximately 10% compared to that before acid leaching (Fig. 3b). It is worth noting that 10.62% of incompletely converted HMFCA was also present in the Co-N_2_ reaction system, and similar phenomena were observed in the Co-N_3_ and Co-N_4_ catalytic systems (the decreased amount of FDCA was basically consistent with the unconverted HMFCA) (Figs. 3c and S10). These findings reveal that the Co_4_ site in the Co-N_2_/Co_4_ catalyst plays a pivotal role in accelerating the oxidation process of the hydroxymethyl side chain in intermediate HMFCA. High conversion efficiencies of HMF and FFCA bearing the aldehyde side chain were consistently observed across all five representative SACs systems. This finding underscores the critical role of single-atom sites in accelerating the oxidation of the aldehyde on the furanic ring.

Remarkably, the catalytic enhancement effect of Co cluster sites exhibits an inverse correlation with increasing cluster particle size. Specifically, the Co-N_2_/Co_6_ catalytic system demonstrates significantly decrease in HMF oxidation efficiency compared to its Co-N_2_/Co_4_ counterpart. To elucidate the distinct roles of Co clusters and Co–N coordination, we employed KSCN poisoning experiments to selectively deactivate Co–N active structure in both catalysts. As evidenced in Fig. S12, Co_6_ clusters display pronounced suppression of HMF-to-HMFCA conversion relative to Co_4_ clusters under equivalent Co–N site inhibition conditions, establishing a size-dependent inhibitory effect. Additionally, the lower HMFCA product in both cluster catalytic systems indicates that Co_4_ clusters and Co_6_ clusters can contribute to accelerating the conversion of HMFCA hydroxymethyl intermediates, which is consistent with the above speculation (Table S5). Remarkably, the degree of catalytic suppression for both HMF conversion and FDCA formation directly correlates with Co–N bond depletion, providing compelling evidence that Co–N active structure predominantly mediates the sequential oxidation of aldehyde groups on the furan ring.

To gain further insight into the catalytic functions of the distinct Co sites, the reaction intermediate HMFCA was employed as the starting substrate in control experiments. Notably, the Co-N_2_/Co_4_ (KSCN) catalyst displayed superior activity in converting HMFCA to FFCA. However, this deficiency in single-atom sites consequently impeded the subsequent FFCA oxidation, leading to a notably low FDCA yield. In stark contrast, the Co-SACs catalyst, while less active in the initial step from HMFCA to FFCA, exhibited exceptional efficiency in driving the critical FFCA-to-FDCA transformation (Fig. S11). According to kinetic regime analyses (Conv. < 10%) at 55 °C, when the reaction starts with HMF or FFCA, Co-N_2_/Co_4_, and Co-N_2_/Co_4_ (etched) SACs, exhibit 2–4 times higher conversion and reaction rate than Co-N_2_/Co_4_ (KSCN) SACs. In contrast, when HMFCA is used as the substrate, Co-N_2_/Co_4_ and Co-N_2_/Co_4_ (KSCN) SACs (with cluster sites) show 2–5 times greater activity in both conversion and rate compared to Co-N_2_/Co_4_ (etched) SACs (Fig. 3d; Table S6). The above experimental results proposed a potential mechanism that single-atom Co site predominantly drive furan-ring aldehyde oxidation to carboxylic acids, while adjacent Co clusters govern selective hydroxymethyl dehydrogenation, thus synergistically achieving stepwise HMF cascade oxidation (Figs. 3e and S13).

**Fig. 4:**
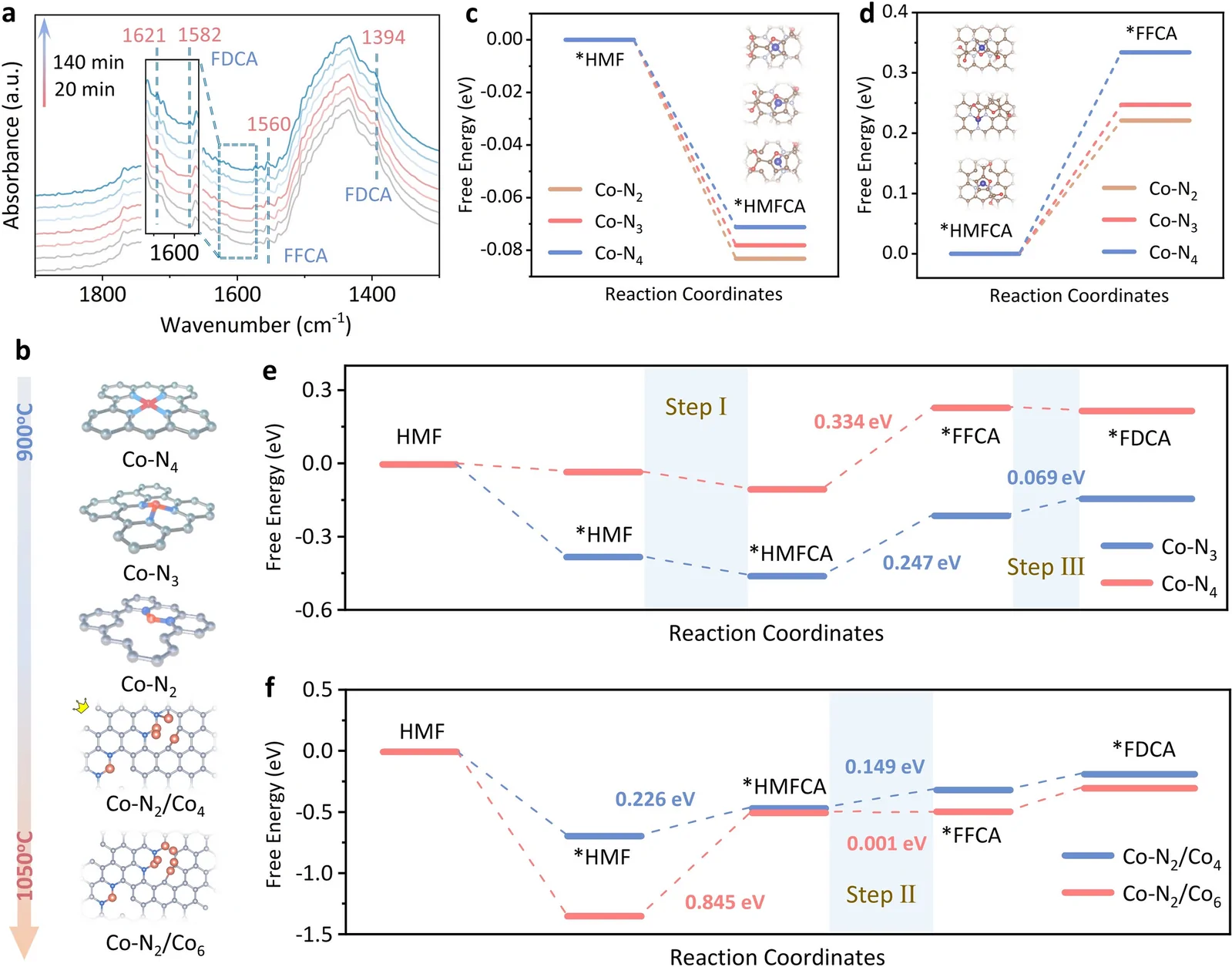
DFT calculations for mechanism investigation. **a** FTIR spectra were collected during HMF oxidation on Co-N_2_/Co_4_ SACs; **b** Catalyst optimization models for the Co catalysts; **c**, **d** Energy barrier of HMF to HMFCA and HMFCA to FFCA over the Co-N_2_, Co-N_3_, Co-N_4_ SACs; **e**, **f** Reaction pathway and corresponding energy of Co catalysts

Moreover, in situ infrared spectroscopy was employed to monitor the temporal evolution of oxidation products, allowing for direct comparison of the HMF oxidation kinetics over different catalysts. Upon prolonging the reaction time, characteristic absorption peaks assignable to FDCA emerged at 1386, 1582, and 1621 cm^−1^ for the Co-N_2_/Co_4_ SACs. Crucially, the intensities of these peaks were significantly higher than those observed for the mono-atomic-site catalyst. These results unequivocally demonstrate the superior HMF oxidation activity of the Co-N_2_/Co_4_ dual-site catalyst, which is in excellent agreement with the experimental findings (Figs. 4a and S14). Notably, the FFCA characteristic peak at 1560 cm^−1^ showed distinct intensification over Co-N_2_/Co_4_ SACs, signaling substantial accumulation of this intermediate. Collectively, these observations confirm that HMFCA oxidation to FFCA is markedly facilitated on the single-atom catalyst featuring cluster sites.

### Theoretical Studies Insights on Catalytic Mechanism

DFT calculations were also used to validate the stepwise enhancement mechanism of Co-N_2_/Co_4_ dual sites in HMF oxidation. Based on the above experiments and previous reports [30], the structural models of Co single-atom and Co single-atom/cluster were rationally constructed. Following geometric optimization, small Co_4_ and Co_6_ nanometric clusters coexisting with single-atom units were identified (Fig. 4b). The bond lengths of Co–N and Co–Co in the catalyst were determined to be approximately 2.38 and 1.92 Å, respectively, which are consistent with the XAS results (Fig. S15; Tables S3 and S4).

First, we systematically evaluated the catalytic oxidation performance of three single-atom active sites for HMF and its oxidized product, HMFCA. As illustrated in Fig. 4c, all single-atom catalytic sites exhibit thermodynamically spontaneous characteristics (ΔG < 0) during the oxidation steps of converting the HMF aldehyde side chain to carboxylic acids. Furthermore, the Co-N_2_ site demonstrates the lowest activation energy barrier (ΔG = −0.083 eV), validating the significant promoting effect of Co-N_2_ active centers on the sequential oxidation of the aldehyde group. Notably, in the terminal oxidation stage from FFCA to FDCA, the energy barrier differences remain constrained below 0.069 eV among all single-atom sites catalysts, demonstrating the absence of kinetic limitations in the catalytic system (Figs. 4e and S16). However, the Co SACs prepared without introducing nanocluster sites displayed substantially increased energy barriers (ΔG > 0.221eV) for the subsequent oxidation of HMFCA to FFCA (Fig. 4d). Theoretical calculations further demonstrate that constructing Co-N_2_/Co_4_ dual-site catalysts reduces the oxidation energy barrier of the HMFCA hydroxymethyl side chain to 0.149 eV. Consequently, the Co_4_ nanocluster effectively promotes the activation of the hydroxymethyl side chain by optimizing the intermediates adsorption configurations.

Crucially, when the Co nanocluster size exceeds a critical threshold (> 4 atoms), although maintaining high activity for hydroxymethyl oxidation (ΔG = 0.001 eV), the steric hindrance effects may compromise the initial activation efficiency of HMF at single-atom sites (Figs. 4f and S17). Both experimental and theoretical results reveal a synergistic catalytic mechanism between Co-N_2_ single-atom sites and Co_4_ nanoclusters, wherein the Co-N_2_ configuration directs selective oxidation of aldehyde groups while adjacent Co_4_ clusters mediate kinetically favorable hydroxymethyl activation. In this dual-site cooperative strategy, single-atom sites ensure high efficiency in the initial reaction step, whereas nanoclusters provide specific intermediate activation functionality. This approach successfully addresses the inherent functional constraints of SACs in complex multi-step oxidation reactions.

### Catalytic Performance for HMF Oxidation Reaction over Co-N_2_/Co_4_ SACs

**Fig. 5:**
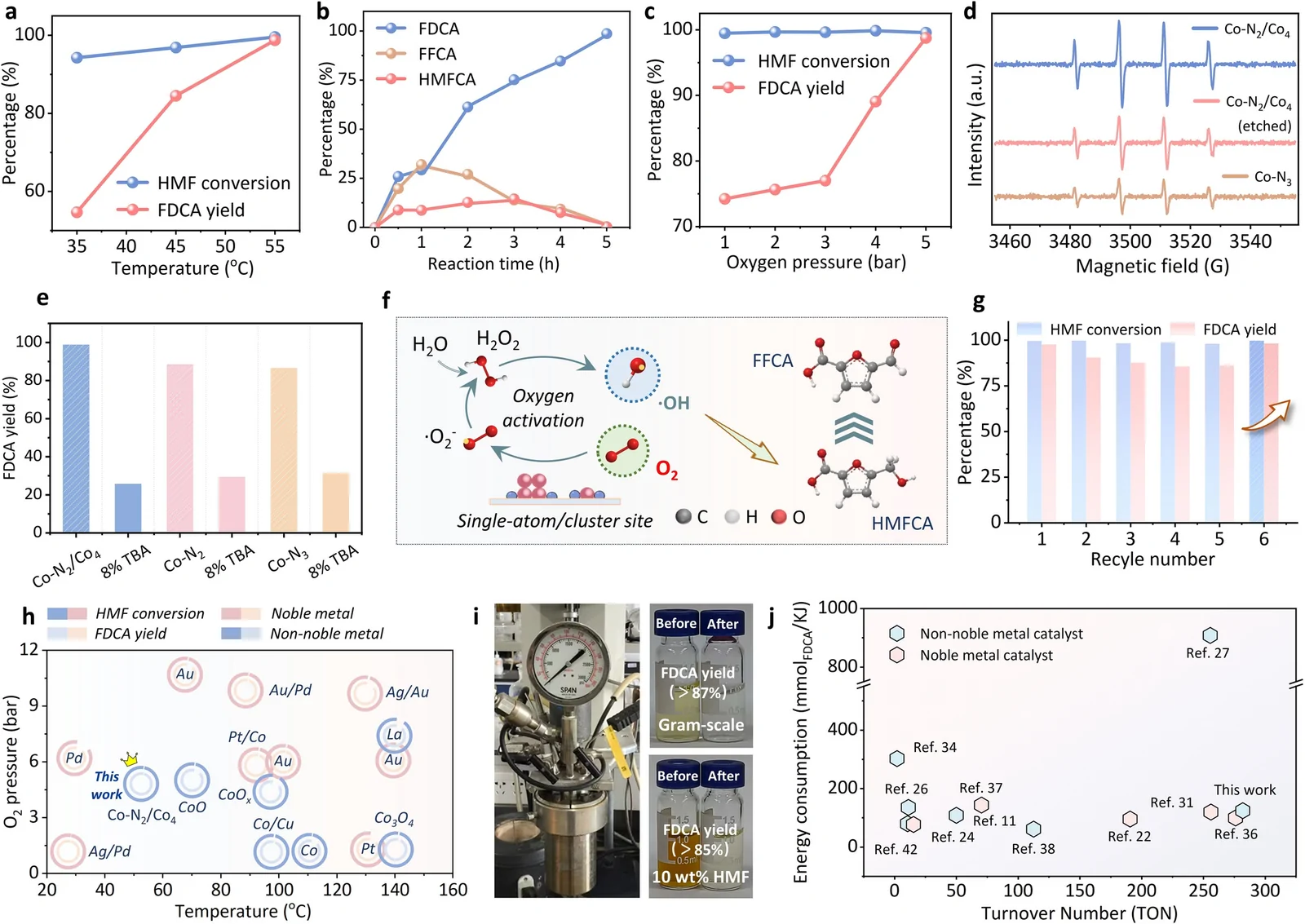
Performance assessment of Co-N_2_/Co_4_ catalyzed HMF oxidation to FDCA. **a** Impacts of reaction temperature, **b** reaction time and **c** oxygen pressure on the HMF catalytic process over the Co-N_2_/Co_4_ SACs; **d** EPR spectra of the Co-N_2_/Co_4_ SACs; **e** Quenching results in Co catalytic system; **f** Oxidation process from HMFCA to FFCA; **g** Recyclability of Co-N_2_/Co_4_ SACs; **h** Statistical data on catalysts for HMF oxidation reactions are presented; **i** Schematic diagram of the stainless-steel reactor and HMF scale-up experiment; **j** Comparison of TON and energy consumption statistics

The various factors of reaction temperature, reaction time, and oxygen pressure for HMF catalytic efficiency over the Co-N_2_/Co_4_ SACs were deeply researched and discussed. The oxidation process of HMF demonstrates pronounced temperature dependence due to the inherent reactivity of its hydroxymethyl and aldehyde functional groups. As depicted in Fig. 5a, within the temperature span of 35–55 °C, the HMF conversion was maintained at a comparably elevated level. Upon a gradual elevation of temperature, the formation of FDCA was notably facilitated. Eventually, the FDCA yield reached 98.76% at 55 °C, thereby attaining a recyclable carbon balance. To better elucidate the oxidation process, the time-dependent behavior of Co-N_2_/Co_4_ SACs monitored. Under mild conditions, dual-active-site Co-N_2_/Co_4_ SACs demonstrated superior catalytic efficiency compared to Co SACs, achieving > 50% HMF conversion within 0.5 h. Kinetic profiling revealed dynamic intermediate evolution HMFCA and FFCA concentrations peaked at 1 ~ 3 h (14.52% and 27.03%, respectively) before gradual depletion, culminating in 98.76% FDCA yield at 5 h (Fig. 5b). In contrast, single-site Co–N configurations retained elevated HMFCA levels (> 10%) even at 5 h (DFT-calculated ΔE = 0.2 ~ 0.35 eV), highlighting the critical role of Co_4_ sites in lowering the activation energy for HMFCA oxidation (Table S7).

In the reaction system of HMF to FDCA, the aldehyde group and hydroxymethyl group in HMF molecules are gradually oxidized to two carboxyl groups with the participation of oxygen [38]. Consequently, the HMF catalytic efficiency is also influenced by the metal active site within the catalyst regarding its oxygen activation ability. As shown in Fig. 5c and Table S8, the dual-site Co-N_2_/Co_4_ SACs activates HMF to generate significantly higher FDCA yields than SACs under identical oxygen pressure. Notably, while both catalytic systems exhibit comparable HMF conversion and residual FFCA levels, the diminished FDCA yield in the single-site system primarily originates from accumulated unconverted HMFCA intermediates. This observation highlights the exceptional HMFCA activation capacity of Co-N_2_/Co_4_ SACs, which is attributed to the oxygen activation enhancement enabled by the introduced Co_4_ site.

Subsequently, EPR spin capture experiments were conducted to verify the enhancement mechanism of HMF catalytic oxidation by Co catalysts using oxygen as oxidant. As illustrated in Fig. 5d, the characteristic radical signal of ·OH was identified in both the single-atom and atom/cluster reaction systems. Nevertheless, in contrast to the single-atom system, the EPR signal intensity in Co-N_2_/Co_4_ catalytic system increased, suggesting that more ·OH is produced during the catalytic reaction. Furthermore, in the absence of oxygen, the EPR signal intensity in the Co catalytic system declined significantly, indicating that ·OH might result from oxygen activation (Fig. S18). Subsequently, tert-butanol (TBA) was utilized as an ·OH scavenger to validate the influence of OH on the HMF catalytic oxidation system [39]. As shown in Fig. 4e, when 8% TBA was added to the catalytic system, the FDCA yield over the three lignin-based Co catalysts decreased by approximately 65%, revealing that ·OH is the key reactive oxygen species in the oxidation of HMF to FDCA. Therefore, the possible activation pathway of oxygen over the catalyst is proposed. Oxygen scavenges the electrons released by OH^−^ at the active site to form O_2_^−^, subsequently, hydrogen peroxide is produced under the influence of an alkaline environment, and then hydrogen peroxide decomposes to generate·OH, which participates in the HMF oxidation process (Fig. 5f). Compared to single-site catalysts, Co-N_2_/Co_4_ SACs are proposed to activate enhanced ·OH generation, accelerating HMFCA oxidation to FFCA.

### Recyclability and Applicability of Co-N_2_/Co_4_ SACs

To verify the reusability of Co-N_2_/Co_4_ SACs in the HMF oxidation, the catalysts were retrieved from the catalytic system by filtration and washing with deionized water, without undergoing any further processing. The HMF conversion exceeded 99% after five continuous reactions under identical conditions. The corresponding FDCA yield remained above 85%, suggesting that the catalyst possesses preferable cyclic stability, which might be attributed to the strong alkali resistance of the Co-N_2_/Co_4_ configuration in the catalytic system. Notably, the catalyst was regenerated in a hydrogen atmosphere (300 °C, 2h) after the fifth reaction cycle. After the regeneration process, the FDCA yield increased by approximately 13% compared with the fifth reaction (Fig. 5g). The primary cause might be that the high temperature treatment eliminates the products, by-products or other impurities adsorbed on the catalyst surface, thereby restoring their catalytic activity [30]. Additionally, TEM and HAADF-STEM images demonstrate the stable retention of single-atoms and clusters in the catalyst after six reaction cycles, with no significant nanoparticle formation, which is further corroborated by XRD analysis of the recycled Co-N_2_/Co_4_ SACs (Figs. S19 and S20). The Co content after reaction was quantitatively analyzed by ICP-MS. The Co content in Co-N_2_/Co_4_ SACs still amounted to 94.11% of the initial content, indicating that the Co-N_2_/Co_4_ SACs present considerable stability (Table S9).

Compared with supported metal catalysts reported in recent years, Co-N_2_/Co_4_ SACs have accomplished the FDCA oriented conversion under low-temperature (55 °C) conditions, especially demonstrating a leading position in the reaction temperature among Co-based catalysts. Although a few supported noble metal catalysts had been reported to catalyze the reaction at lower temperatures and oxygen pressures, large-scale applications of these catalysts may be constrained by their higher catalytic costs (Fig. 5h; Table S10) [40–56]. Gram-scale HMF oxidation at 55 °C achieved an instantaneous FDCA production rate of 141.6 mol_FDCA_ mol_Co_^−1^ h^−1^ within 5 min, reaching > 87% yield after 5 h. For industrial-concentration adaptation (10 wt% HMF), Co-N_2_/Co_4_ SACs maintained > 85% FDCA yield over 12 h despite prolonged reaction time due to mass transfer constraints (Figs. 5i and S21). Subsequently, the catalyst-free supernatant (obtained via centrifugation) was acidified to pH 1–3 to trigger FDCA crystallization, further confirming the industrial scalability of Co-N_2_/Co_4_ SACs [57]. Moreover, the turnover number (TON) of Co-N_2_/Co_4_ SACs and the energy consumption of FDCA production are preferable compared with those of the metal-supported catalysts reported (Figs. 5j and S22).

The emergence of FDCA as a promising biomass-based alternative to petroleum-derived terephthalic acid (PTA) necessitates a systematic comparative assessment of its environmental and economic performance. First, a life cycle assessment (LCA) was conducted to compare the Co dual-site thermocatalysis (CDST) process with the conventional petroleum-derived PTA counterpart. Using 1 kg FDCA as the functional unit, the system boundary encompasses raw material production, catalyst preparation, and reaction processes (Table S11). The results demonstrate that CDST process achieves a significant reduction of 82.47% in fossil fuel potential and exhibits notable environmental advantages in categories such as freshwater eutrophication, ecotoxicity, and human toxicity potential. In contrast, the two pathways exhibit comparable impacts in terms of global warming and terrestrial acidification potential, with the primary influencing factors originating from the energy consumption and complexity associated with HMF production (Fig. S23). Meanwhile, compared with reported systems, the CDST process offers a more sustainable catalytic pathway due to its significantly lower global warming potential. Furthermore, a preliminary techno-economic analysis (TEA) indicates that the economic viability of this process heavily depends on the cost of the feedstock HMF, which accounts for approximately 50%–60% of the total expenses (Fig. S24 and Note S1). Under industrial-scale scenarios, the current process already demonstrates considerable profit potential. Future efforts should focus on optimizing upstream processes to substantially reduce the HMF cost, thereby significantly enhancing overall economic competitiveness.

**Fig. 6:**
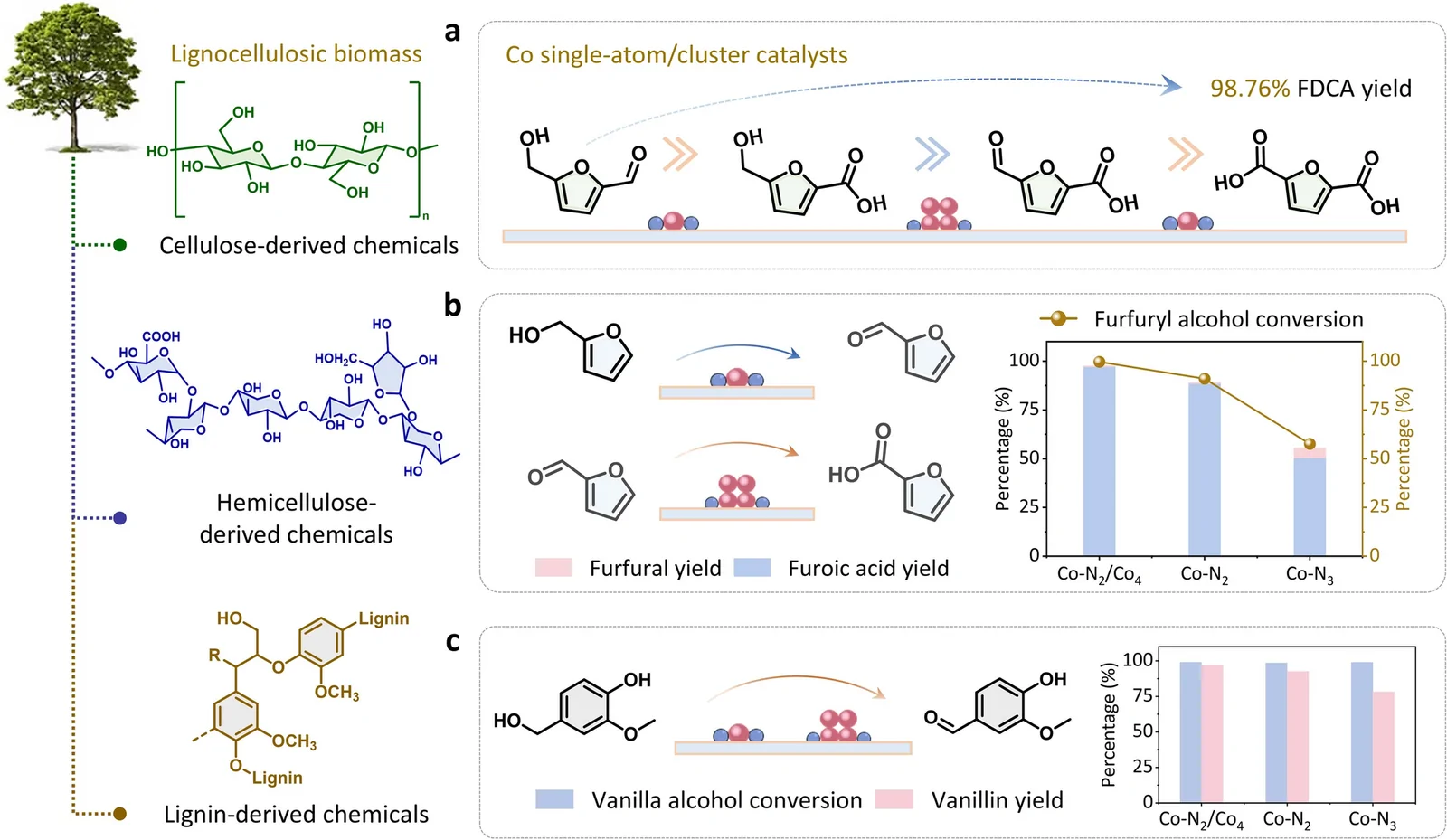
High selective oxidation of lignocellulosic biomass hydroxymethyl platform compounds over the Co-N_2_/Co_4_ SACs. **a**, **b** HMF and furfuryl alcohol are oxidized to FDCA and furoic acid; **c** Vanillin alcohol is oxidized to vanillin

To directly validate the catalytic enhancement effect of the Co₄ cluster sites on the oxidation of hydroxymethyl to aldehyde groups and their functional complementarity with the Co-N_2_ sites, this study additionally employs two representative lignocellulosic biomass-derived platform molecules: furfuryl alcohol and vanillyl alcohol [58, 59]. Among them, the oxidation of furfuryl alcohol (containing a furan ring and a hydroxymethyl group), targeting furoic acid as the final product, is specifically designed to assess the holistic efficacy of both Co_4_ and Co-N_2_ sites in driving the sequential conversion of hydroxymethyl to aldehyde and eventually to carboxylic acid. The oxidation process of vanillyl alcohol to vanillin is uniquely employed to exclusively investigate the catalytic enhancement effect of the Co_4_ cluster sites within the single-atom catalyst on the hydroxymethyl-to-aldehyde conversion. As shown in Figs. 6a and S25, the Co-N_2_/Co_4_ SACs exhibits the highest catalytic performance among the three evaluated catalysts. Upon selective removal of Co_4_ clusters from the Co-N_2_/Co_4_ dual sites via acid-etching treatment (Co-N_2_ SACs), the furoic acid yield decreases by 8.69%. Notably, no significant change is observed in the concentration of the intermediate furfural before and after acid etching of the Co-N_2_/Co_4_ SACs, indicating that the reduced yield primarily stems from incomplete conversion of furfuryl alcohol (Table S12). This confirms the yield loss originates primarily from impaired conversion efficiency in the hydroxymethyl oxidation step due to the absence of Co_4_ sites. Furthermore, complete conversion of furfural is observed over both the pristine Co-N_2_/Co_4_ SACs and its acid-etched counterpart, confirming the highly selective catalytic function of the Co-N_2_ sites toward oxidation of the aldehyde group to carboxylic acid. In contrast, the low reactivity of the Co-N_3_ SACs sample reveals that their restricted catalytic performance originates from the absence of Co_4_ clusters coupled with the higher energy barrier associated with hydroxymethyl activation at the Co-N_3_ sites (Fig. 4c). In addition, in the vanillyl alcohol oxidation system specifically designed to isolate the hydroxymethyl-to-aldehyde conversion pathway, the dual-site Co-N_2_/Co_4_ SACs delivers optimal catalytic performance, achieving > 99% vanillyl alcohol conversion and 97.18% vanillin yield within 1 h (Figs. 6b and S26). These results unambiguously establish the Co_4_ cluster sites as highly efficient catalytic activators for the selective oxidation of hydroxymethyl to aldehyde groups.

## Conclusions

In summary, a “stepwise N-stripping” strategy is developed, leveraging the lignin-derived N-doped carbon matrix as the support to directionally evolve Co active sites from isolated single atoms to atom-cluster hybrid configurations via precisely controllable pyrolysis. This strategy exploits the progressive depletion of N species within the lignin-derived carbon framework, and achieves local modulation of the coordination environment of Co centers during different stages. Combined experimental and theoretical studies demonstrate that single-atom active sites formed in the initial pyrolysis stage selectively activate aldehyde groups, sequential the construction of Co_4_ clusters facilitate hydroxymethyl oxidation through enhanced oxygen activation. The resulting dual-site catalyst integrating atomic and cluster configurations achieves efficient low-temperature cascade oxidation of HMF to FDCA, delivering a 98.76% yield. This work elucidates a synergistic tandem mechanism involving single-atoms and clusters for multi-step processes, advancing biomass valorization and sustainable production of green chemicals through integrated theoretical and technological frameworks.

## Supplementary Information

Below is the link to the electronic supplementary material.

Supplementary file1 (DOCX 3461 KB)
