# Supplementary material for: Ligand-Wise Stripping Dictates Metal Ensemble Catalysts for Selective Oxidation of Biomass-Derived 5-Hydroxymethylfurfural
Source: Nanomicro Lett. 2026 Mar 23;18:299. doi: 10.1007/s40820-026-02118-7 (PMC13009438; doi:10.1007/s40820-026-02118-7)
Supplement: Supplementary file 1 — Supplementary file1 (DOCX 3461 KB) [file 40820_2026_2118_MOESM1_ESM.docx]

Supporting Information for

**Ligand-Wise Stripping Dictates Metal Ensemble Catalysts for Selective Oxidation of Biomass-Derived 5-Hydroxymethylfurfural**

Junkai Li^1^, Guanhua Wang^1,*^, Yunxiang Wu^1^, Chuqiao Song^2^, Tairan Pang^1^, Zechao Zhuang^3^, Jiarui Yang^3^, Wenjie Sui^4,*^, Lili Lin^2,*^, Dingsheng Wang^3^, Ligang Wang^5,*^ and Chuanling Si^1,*^

^1^ State Key Laboratory of Bio-based Fiber Materials, Tianjin Key Laboratory of Pulp and Paper, College of Light Industry Science and Technology, Tianjin University of Science and Technology, Tianjin 300457, P. R. China

^2^ Institute of Industrial Catalysis, State Key Laboratory of Green Chemistry Synthesis Technology, College of Chemical Engineering, Zhejiang University of Technology, Hangzhou 310014, P. R. China

^3^ Department of Chemistry, Tsinghua University, Beijing 100084, P. R. China

^4^ State Key Laboratory of Food Nutrition and Safety, College of Food Science and Engineering, Tianjin University of Science and Technology, Tianjin 300457, P. R. China

^5^ Institute of Molecular Plus, Tianjin University, Tianjin 300072, P. R. China

*Corresponding authors. E-mail: [ghwang@tust.edu.cn](mailto:ghwang@tust.edu.cn) (Guanhua Wang); [wjsui@tust.edu.cn](mailto:wjsui@tust.edu.cn) (Wenjie Sui ); [linll@zjut.edu.cn](mailto:linll@zjut.edu.cn) (Lili Lin); [lgwang_246@tju.edu.cn](mailto:lgwang_246@tju.edu.cn) (Ligang Wang); [sichli@tust.edu.cn](mailto:sichli@tust.edu.cn) (Chuanling Si)

**S1 Materials**

Enzymatic hydrolysis lignin (EHL) utilized for catalyst manufacture was extracted from the enzymatic hydrolysis residue of steam-exploded maize stalk, asper our previous study. 5-Hydroxymethylfurfural (HMF, C_6_H_6_O_3_), 5-hydroxymethyl-2-furancarboxylic acid (HMFCA, C_6_H_6_O_4_), 2,5-furandicarboxylicacid (FDCA, C_6_H_4_O_5_), furan-2,5-dicarbaldehyde (DFF, C_6_H_4_O_3_), 5-formyl-2-furoic acid (FFCA, C_6_H_4_O_4_), 2-furaldehyde, (C_5_H_4_O_2_), 2-furoic acid, (C_5_H_4_O_3_), anhydrous zinc chloride (ZnCl_2_), cobalt chloride hexahydrate (CoCl_2_·6H_2_O), melamine (C_3_H_6_N_6_), sodium carbonate (Na_2_CO_3_), and sodium hydroxide (NaOH) were used.

## S2 Characterization

TEM images were obtained by JEOL JEM-2100 with an acceleration voltage of 200 kV. High angle annular darkfield scanning transmission electron microscopy (HAADF-STEM) images were acquired on Titan cubed 60-300 (FEI, USA) equipped with a probe spherical aberration corrector. The X-ray absorption fine structure (XAFS) spectrum was composed of two distinct components, namely the X-ray absorption near-edge structure (XANES) and the extended X-ray absorption fine structure (EXAFS). XAFS measurements were taken at the 14B2 beam line of Spring-8, Japan’s synchrotron Radiation Facility. The XANES and EXAFS data were processed and analyzed using the ICOFFIT package. X-ray diffraction (XRD) determination of the catalysts was carried out using Rigaku Ru-200b X-ray diffractometer with monochromatic Cu Kα radiation in the scanning range of 5^o^-90^o^. X-ray photoelectron spectroscopy (XPS) was collected by PHI 5000C ESCA spectrometer with Mg Kα radiation (hν 1253.6 eV), and the binding energy was corrected by C 1s value of 284.6 eV. Inductively coupled plasma mass spectrometry (ICP-MS, Thermo Scientific ICAPQ) was employed for quantitative determination of Ni content, and the catalyst was digested using HNO_3_ prior to testing. Fourier transform infrared (FT-IR) measurements were made using a Nicolet Nexus spectrometer.

**S3 Metal-free nitrogen-doped lignin-derived carbon-based catalyst (NC) preparation**

EHL (0.6 g) and melamine (9 g) were uniformly ground, the resultant solid sample was heated in a tube furnace under a nitrogen atmosphere at 550 ^o^C and 900 ^o^C for 1 h respectively. After the completion of pyrolysis, no further treatment was necessary, and subsequent experiments were carried out directly.

**S4 Oxidize gram-scale HMF over the Co catalysts**

The catalytic oxidation of HMF was performed in a 300 mL stainless steel reactor containing 10 g/L HMF, 1mol/L Na_2_CO_3_, 3.5 g Co-N_2_/Co_4_ catalysts, and 150 mL deionized water. The reaction conditions were 5 bar O_2_, 5 h and 55 ^o^C.

**S5 Oxidize high-concentration (10 wt%) HMF over the Co catalysts**

The catalytic oxidation of HMF was performed in a 300 mL stainless steel reactor containing 10 wt.% HMF, 0.1mol/L Na_2_CO_3_, Co-N_2_/Co_4_ catalysts (HMF/Co molar ratio = 65.6), and 150 mL deionized water. The reaction conditions were 10 bar O_2_, 12 h and 120 ^o^C.

**S6 Oxidize furfuryl alcohol to furoic acid over the Co catalysts**

The catalytic oxidation of HMF was performed in a 50 mL stainless steel reactor containing 0.2 mmol furfuryl alcohol, 1M Na_2_CO_3_, 50 mg Co catalysts, and 20 mL deionized water. The reaction conditions were 3 bar O_2_, 5 h and 65 ^o^C. After the reaction, the contents of furfural and its furoic acid in the samples were analyzed by HPLC using Aminex®HPX-87H column (300 mm×7.8 mm) and sulfuric acid solution (5mM) as mobile phase. The contents of furfuryl alcohol in the samples were analyzed by HPLC using C18 column (250 mm×4.6 mm).

**S7 Oxidize vanillin alcohol to vanillin over the Co catalysts**

The catalytic oxidation of HMF was performed in a 50 mL stainless steel reactor containing 0.1 g vanillin alcohol, 0.2g NaOH, 50 mg Co catalysts, and 20 mL deionized water. The reaction conditions were 5 bar O_2_, 1 h and 100 ^o^C. The contents of vanillin alcohol and vanillin in the samples were analyzed by HPLC using C18 column (250 mm×4.6 mm).

## S8 Life Cycle Assessment (LCA) analysis

Analysis is conducted in open LCA 2.0, utilizing its database to identify key environmental impact factors. Employing a “cradle-to-gate” system boundary with 1 kg of FDCA production as the functional unit, the analysis utilized OpenLCA software for modeling. Life Cycle Impact Assessment combines results from the life cycle inventory using the ecoinvent-ReCiPe 2016 v1.03 method, which assesses environmental impact.

**S9 Density functional theory (DFT) calculation**

All the DFT calculations were conducted based on the Vienna Ab initio Simulation Package (VASP) [S1, S2]. The exchange-correlation potential was described by the Perdew-Burke-Ernzerhof (PBE) generalized gradient approach (GGA) [S3]. The electron-ion interactions were accounted by the projector augmented wave (PAW). All DFT calculations were performed with a cut-off energy of 400 eV, and the Brillouin zone was sampled using a 3×3×1 k-point grid. The energy and force convergence criteria of the self-consistent iteration were set to 10^-5^ eV and 0.02 eV Å-1, respectively. DFT-D3 method was used to describe van der Waals interactions [S4].

The Gibbs free energy changes (Δ*G*) of the reaction are calculated using the following formula:

∆*G* = ∆*E* + ∆ZPE - T∆*S*

where Δ*E* is the electronic energy difference directly obtained from DFT calculations, ΔZPE is the zero-point energy difference, *T* is the room temperature (298.15 K) and ΔS is the entropy change.

The adsorption energies (*E*ads) of a molecule are calculated by

*E*_ads_ = *E**_A_–*E*_A_ –*E*_Sub_

where *E*_A_ and *E**_A_ represent the energies before and after the adsorption of a molecule on the substrate, respectively. *E*_sub_ is the energy of clean surface.

**Supplementary Figures and Tables**


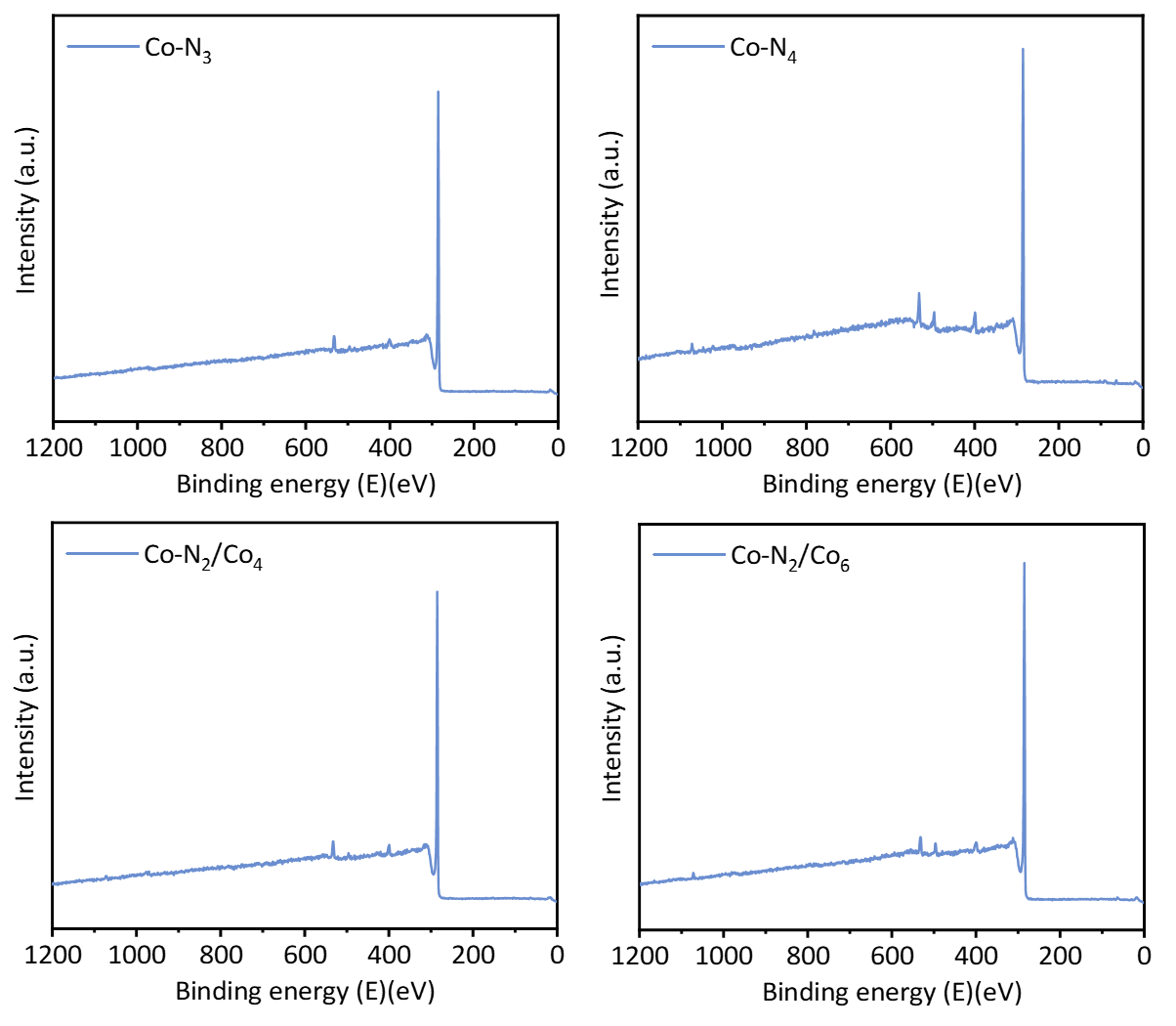


**Fig.** **S1** XPS spectra of Co catalysts


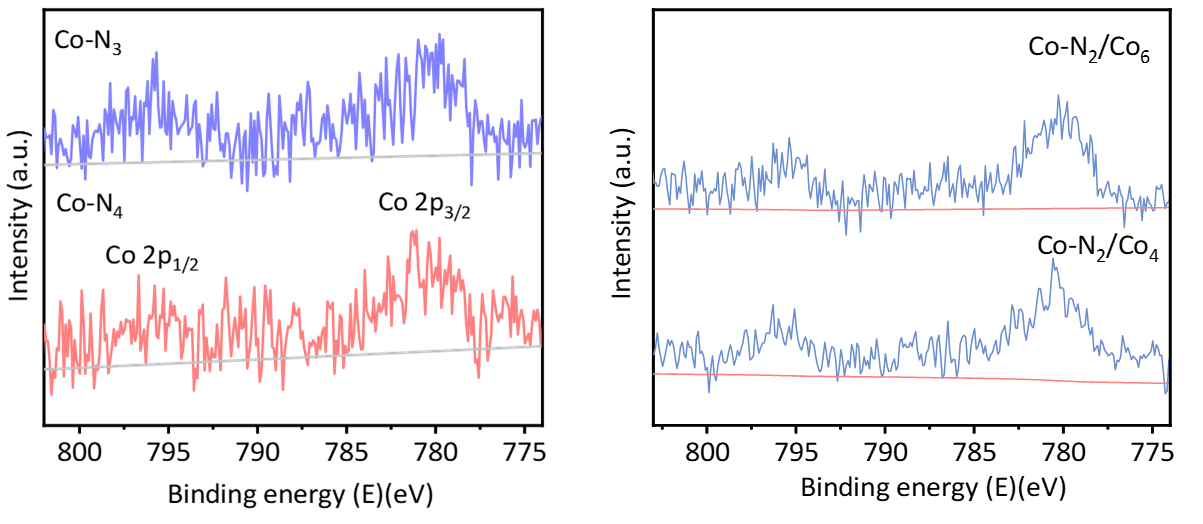


**Fig. S2** Co 2p XPS spectra of Co catalysts


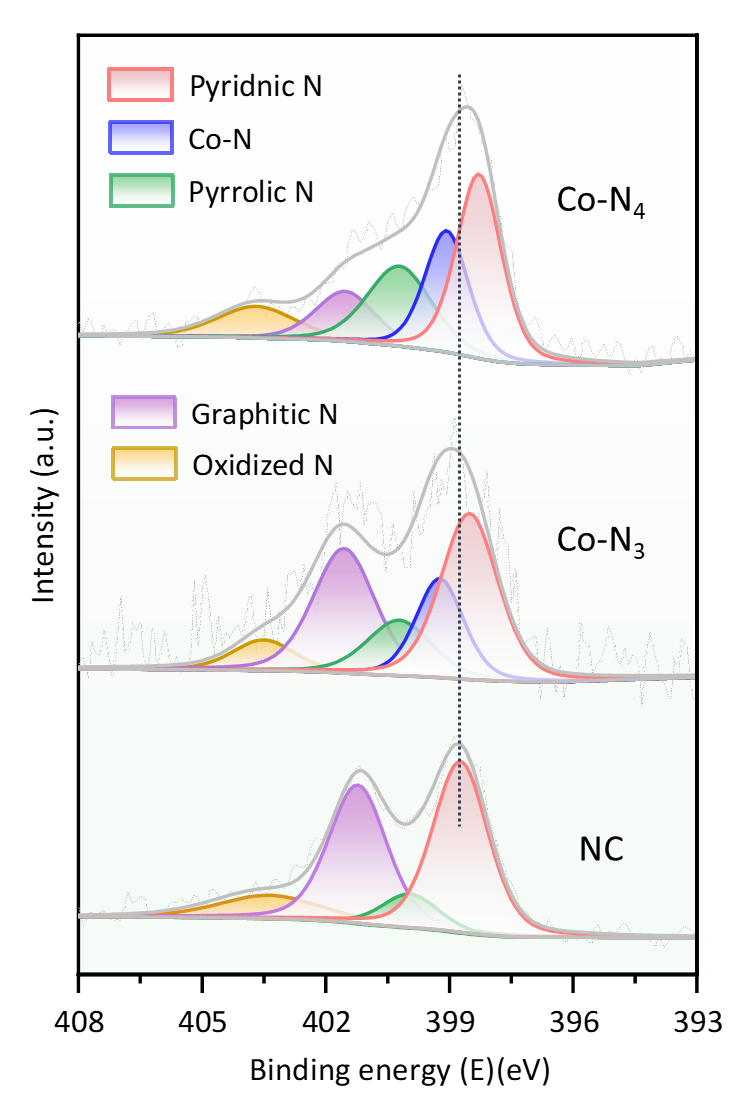


**Fig. S3** N 1s XPS spectra of Co catalysts and NC catalysts

**Fig. S4** XANES and EXAFS spectra of the Co samples


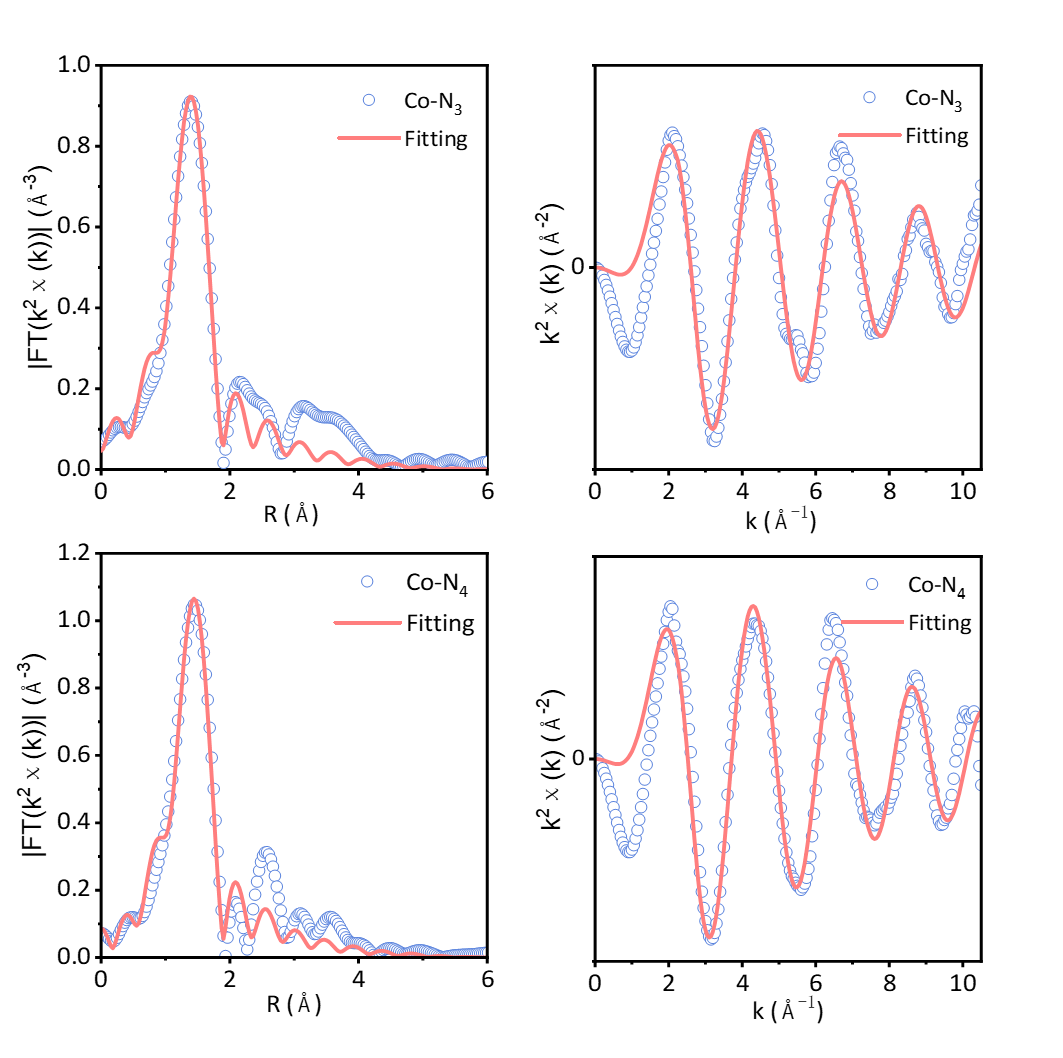


**Fig. S5** EXAFS fitting curves of Co-N_3_ SACs and Co-N_4_ SACs at R space and K space


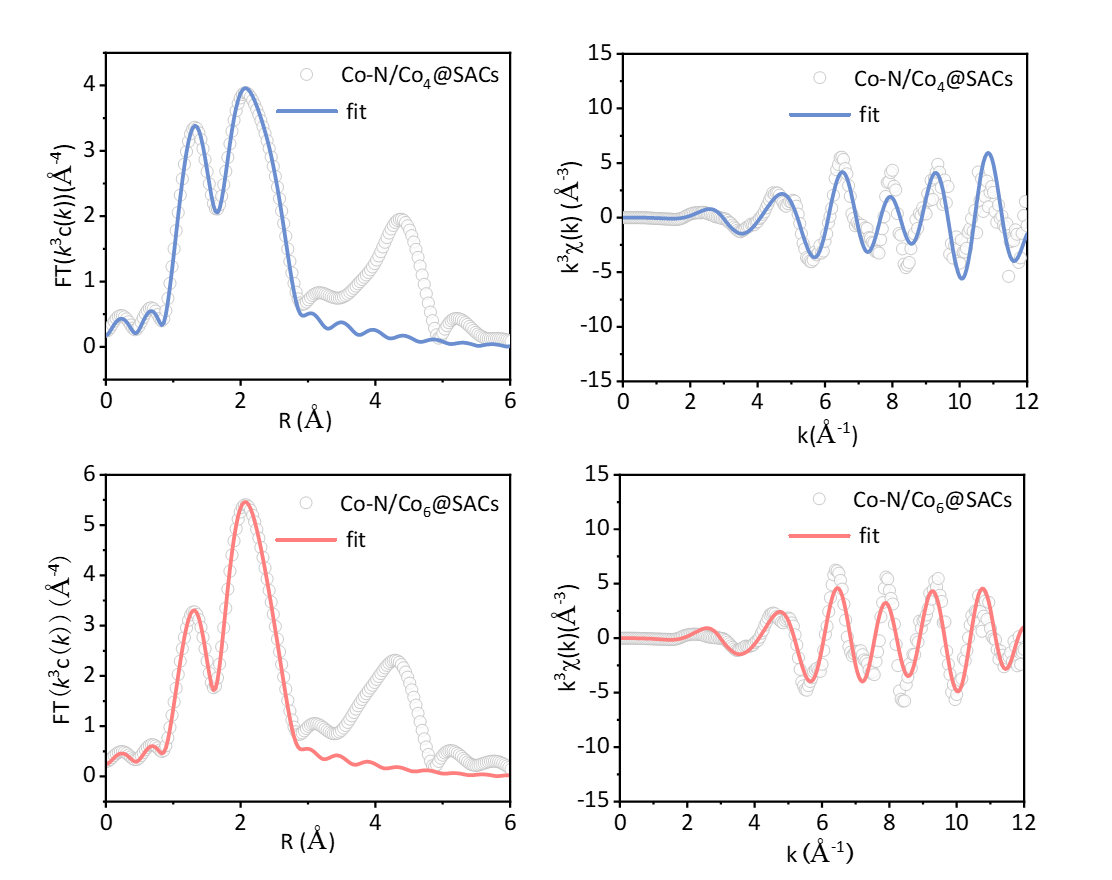


**Fig. S6** EXAFS fitting curves of Co-N_2_/Co_4_ SACs and Co-N_2_/Co_6_ SACs at R space and K space


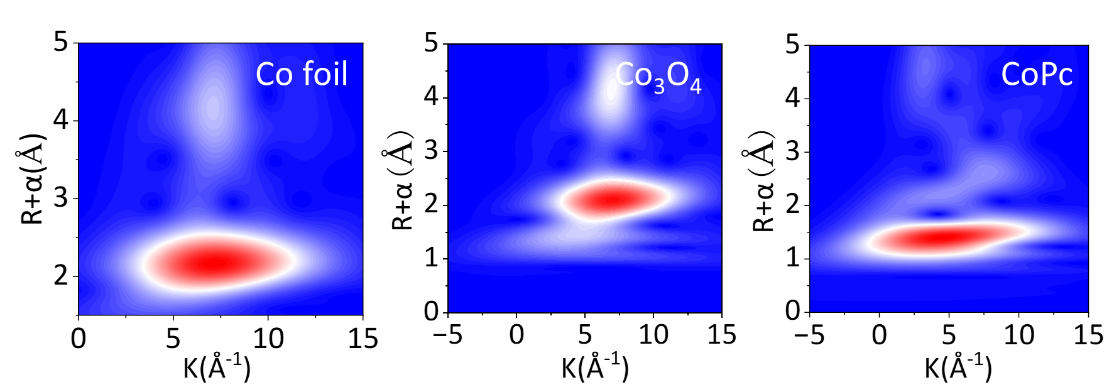


**Fig. S7** WT-EXAFS of Co samples
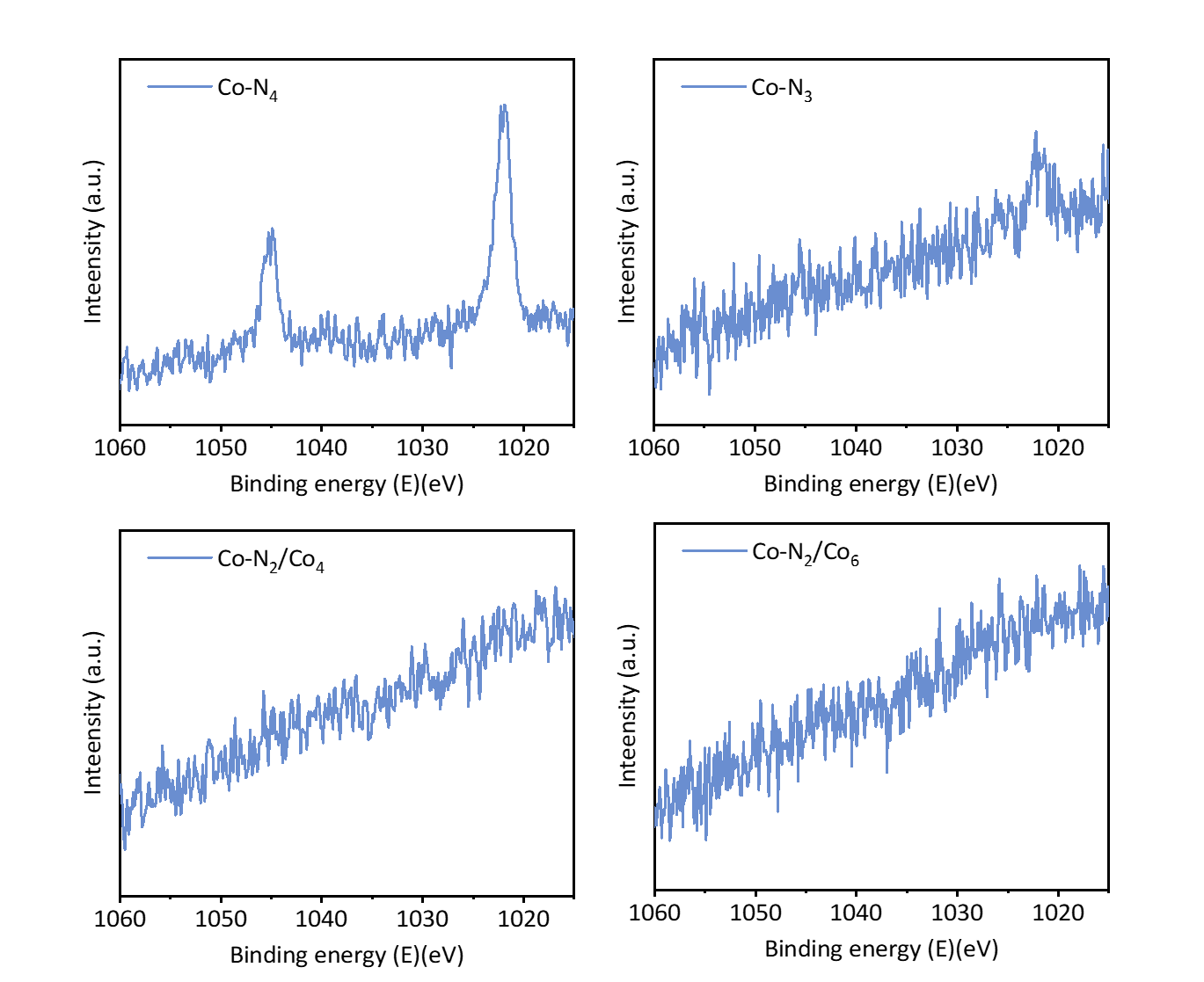


**Fig. S8** Zn 2p XPS spectra of Co catalysts


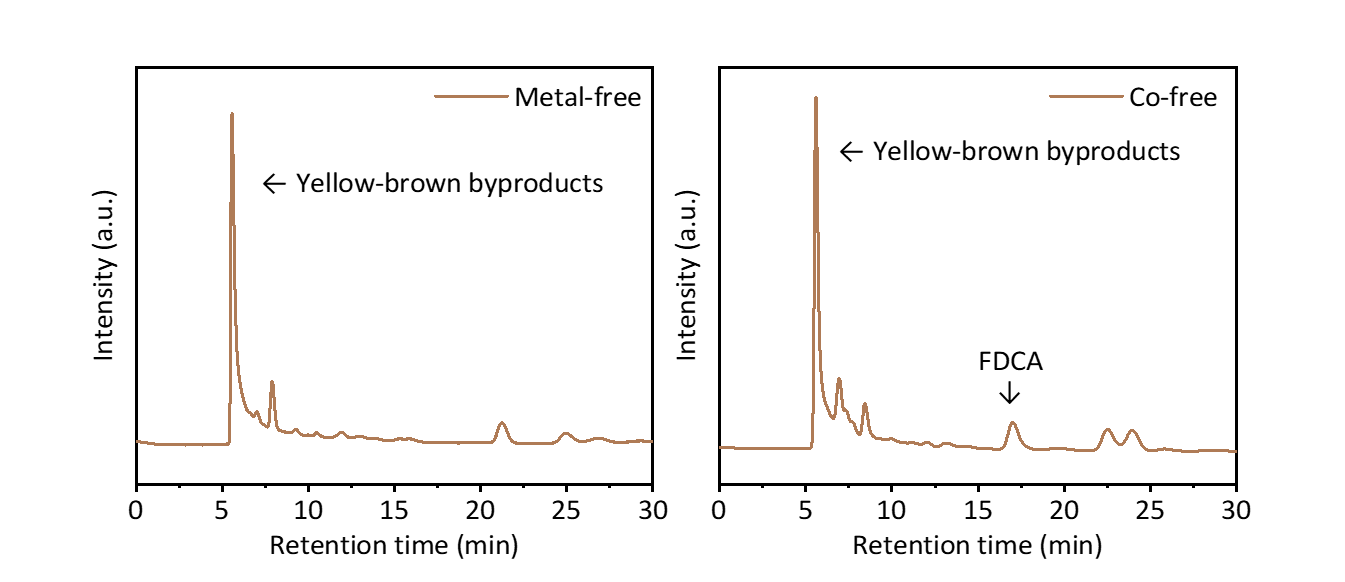


**Fig. S9** HPLC chromatograms of catalytic products


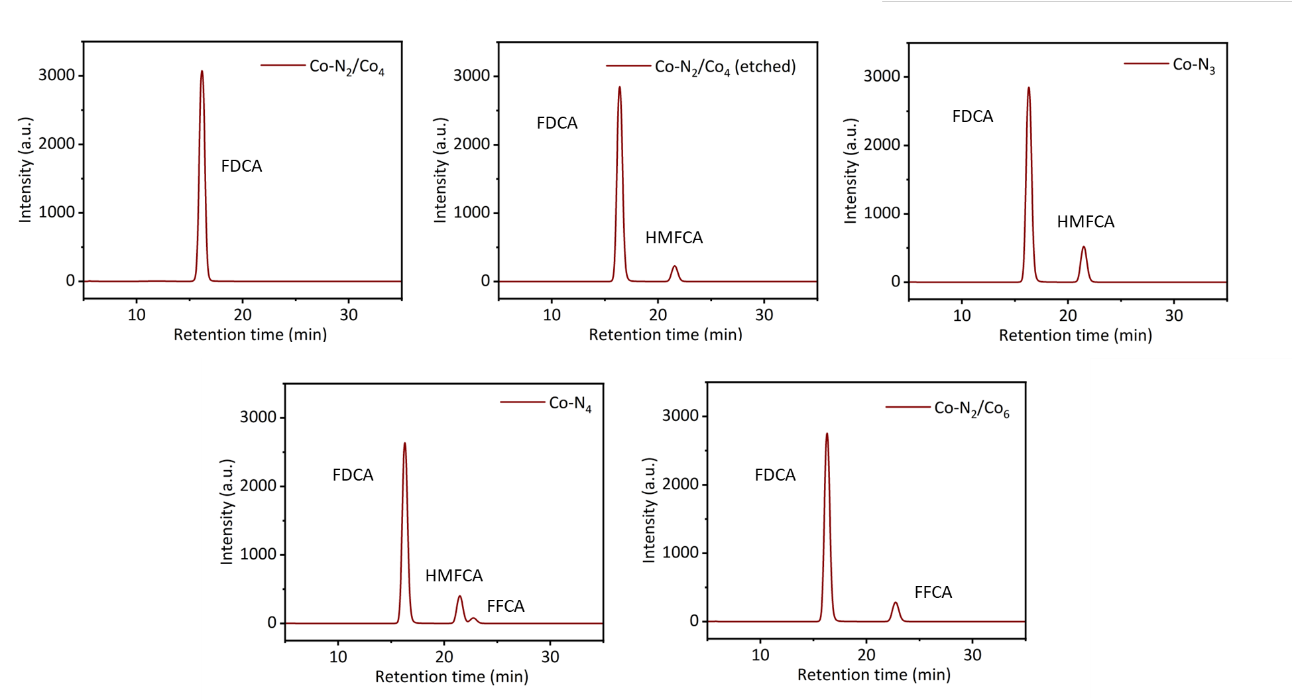


**Fig. S10** HPLC chromatograms of HMF to FDCA


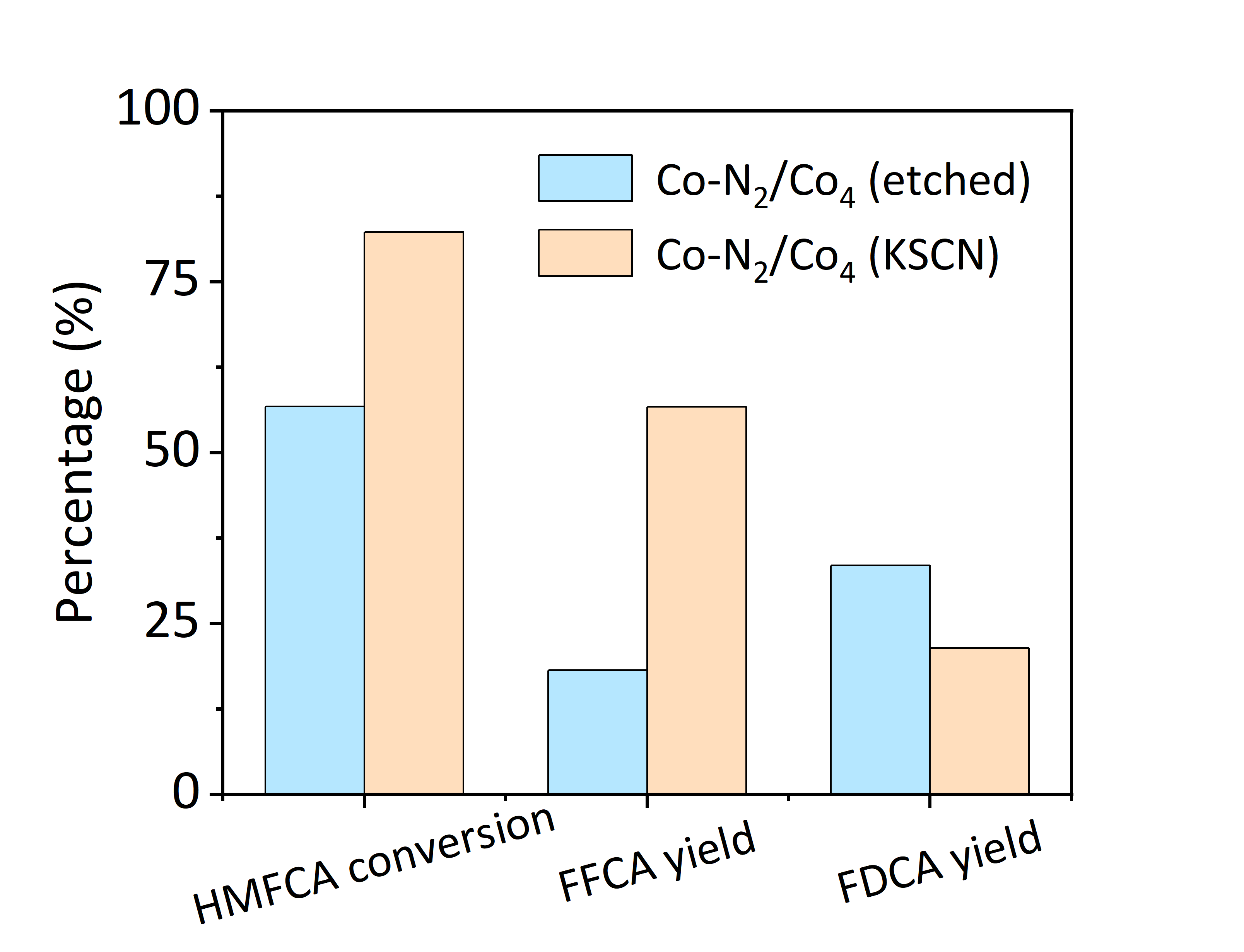


**Fig. S11** Catalytic performance of Co-N_2_/Co_4_ (etched) and Co-N_2_/Co_4_ (KSCN) catalysts for HMFCA to FDCA. Reaction conditions: 5 bar O_2_, 5 h and 55 ^o^C


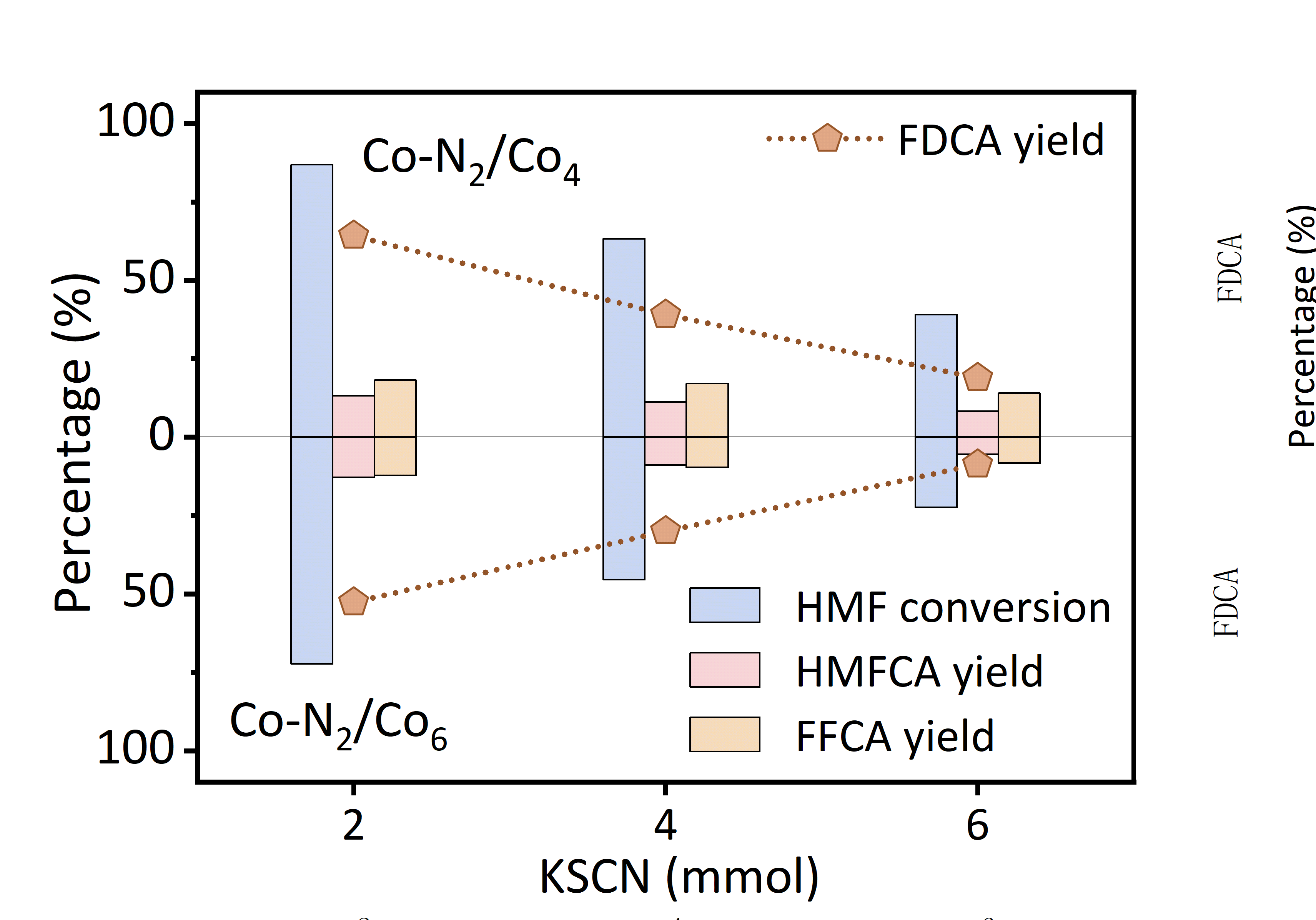


**Fig. S12** The catalytic effect of the Co cluster was verified by poisoning the Co-N_2_ sites with KSCN


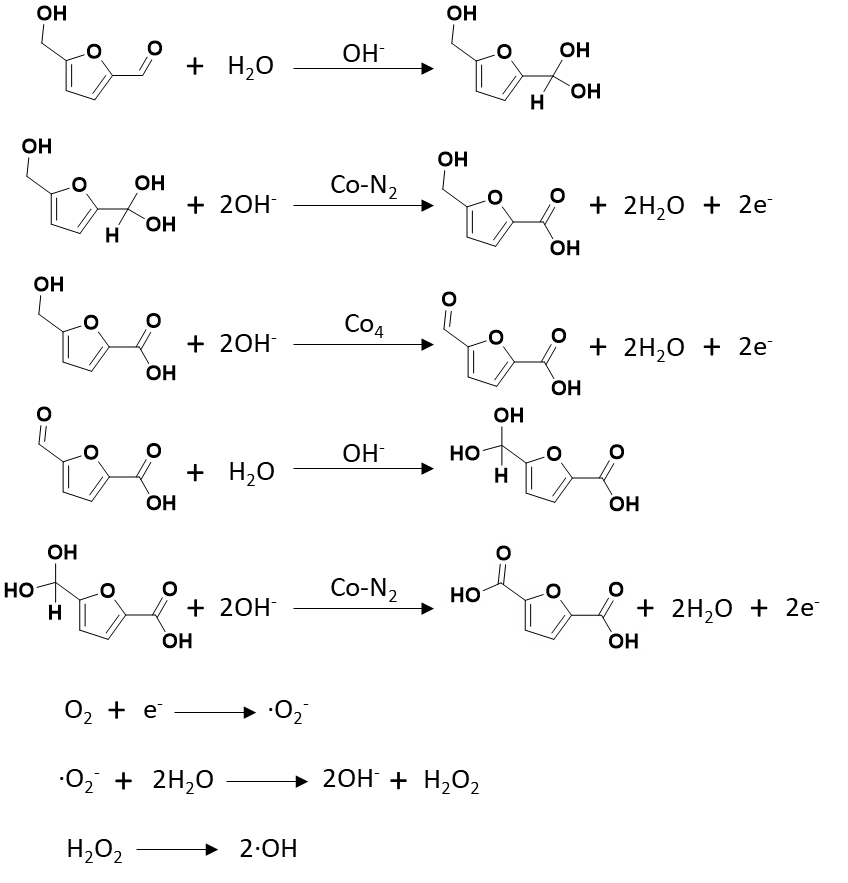


**Fig. S13** Overall mechanism of HMF oxidation to FDCA over the Co-N_2_/Co_4_ SACs


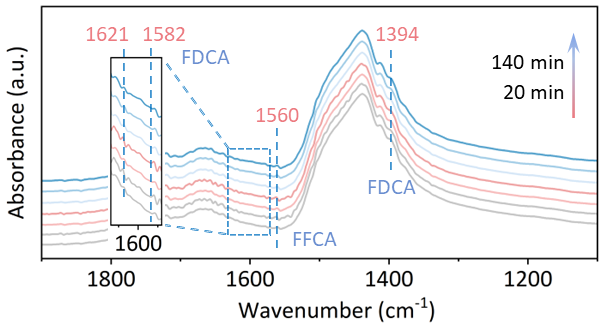


**Fig. S14** FTIR spectra were collected during HMF oxidation on Co single-atom catalyst


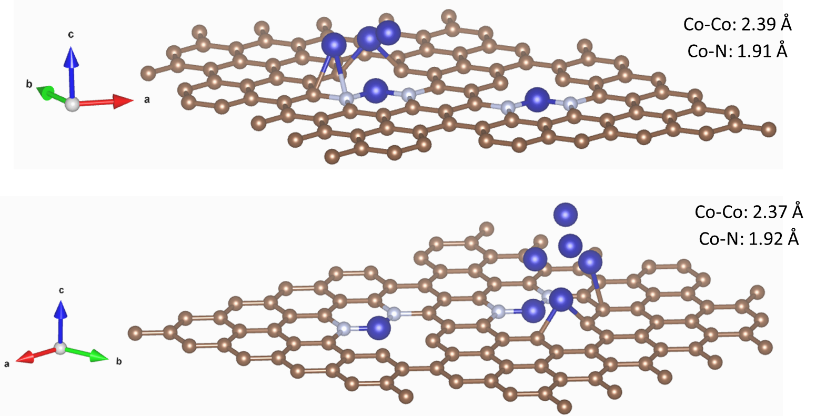


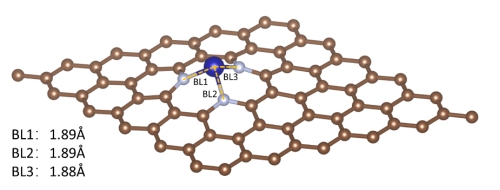

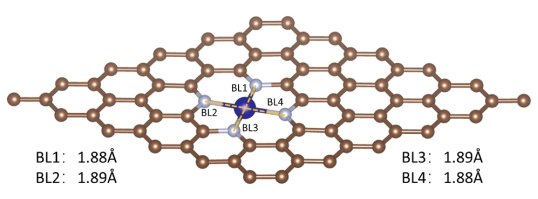


**Fig. S15** The bond length of different bonds in catalyst model


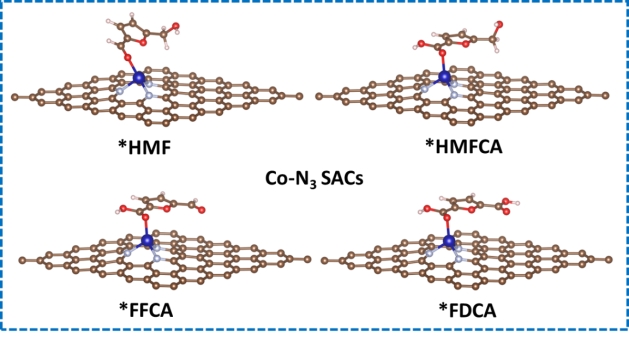


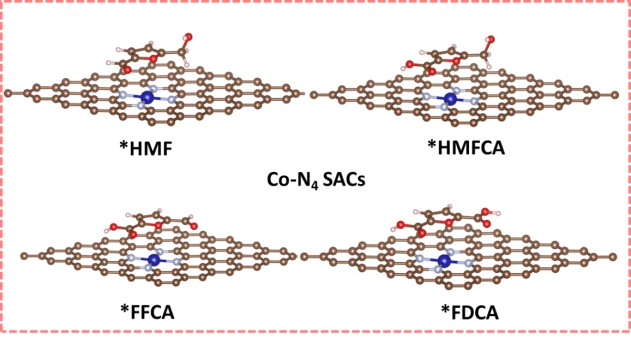


**Fig. S16** HMF dehydrogenation/oxygenation process over the Co-N_3_ SACs and Co-N_4_ SACs


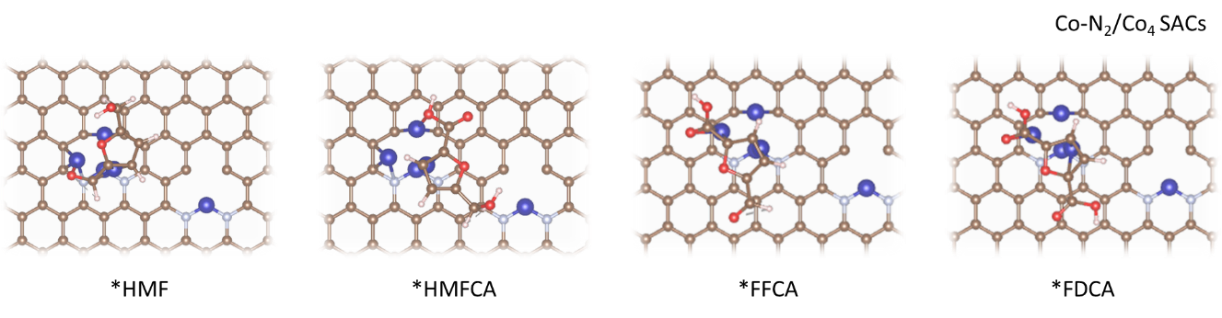


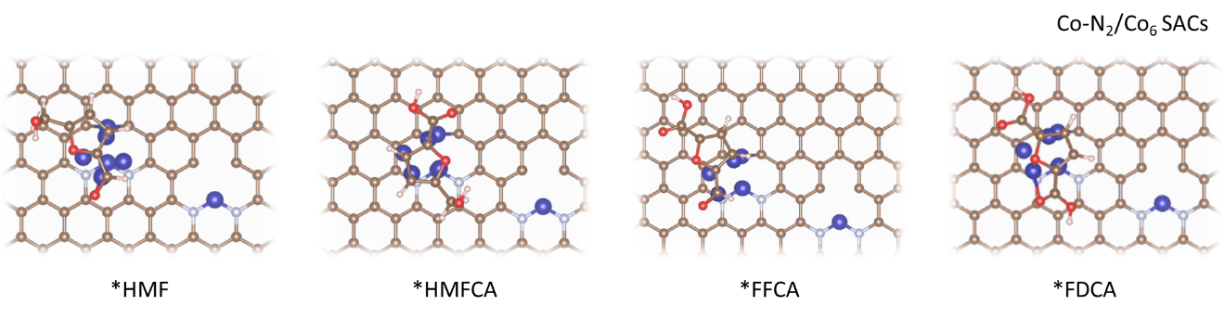


**Fig. S17** HMF dehydrogenation/oxygenation process over the Co-N_2_/Co_4_ SACs and Co-N_2_/Co_6_ SACs


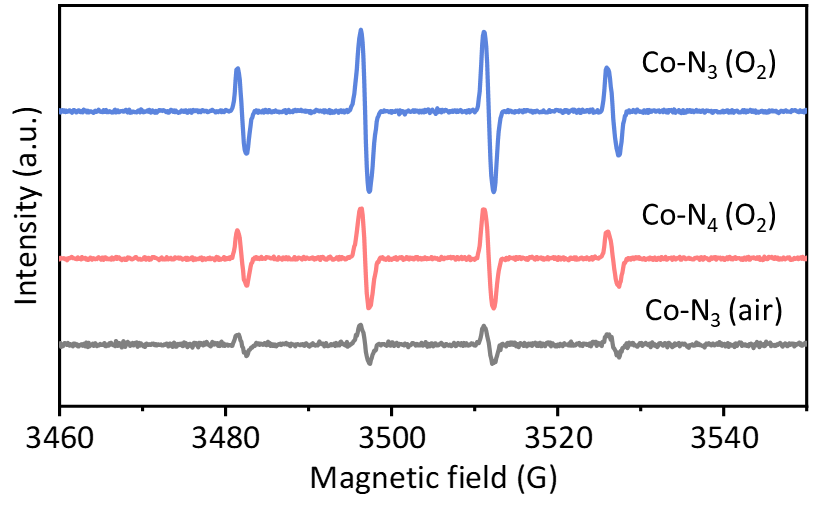


**Fig. S18** EPR spectra of the Co catalysts under different conditions
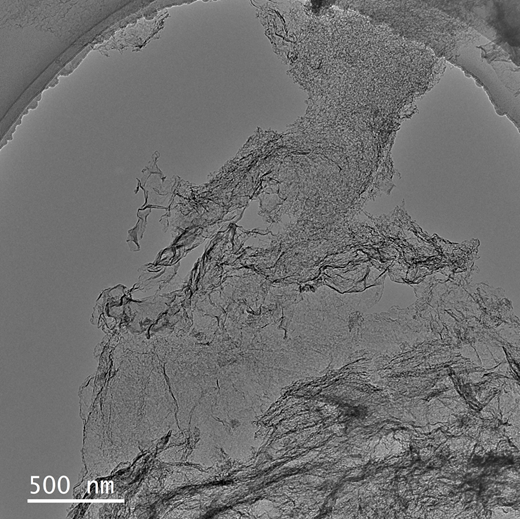

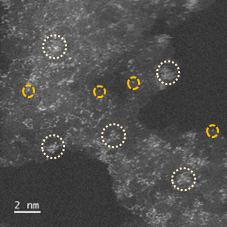


**Fig. S19** HAADF-STEM and TEM images of used Co-N_2_/Co_4_ SACs after six cycles


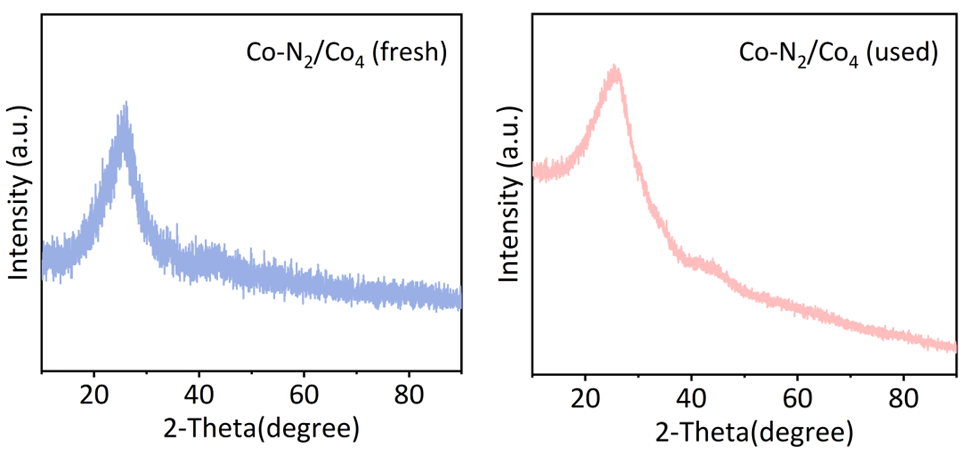


**Fig. S20** XRD spectra of fresh and used Co-N_2_/Co_4_ SACs


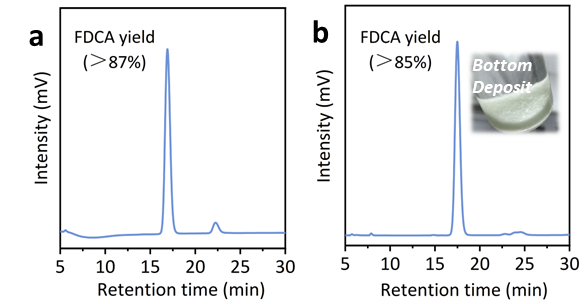


**Fig. S21** (**a, b**) HPLC chromatogram of HMF gram-scale experiment and 10 wt.% HMF concentration experiment


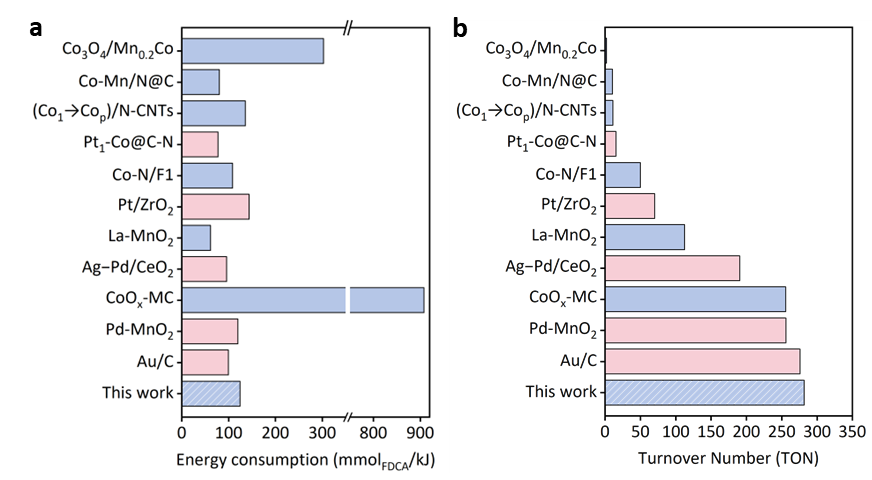


**Fig. S22** (**a, b**) Comparison of energy consumption and TON values between this work and literature data


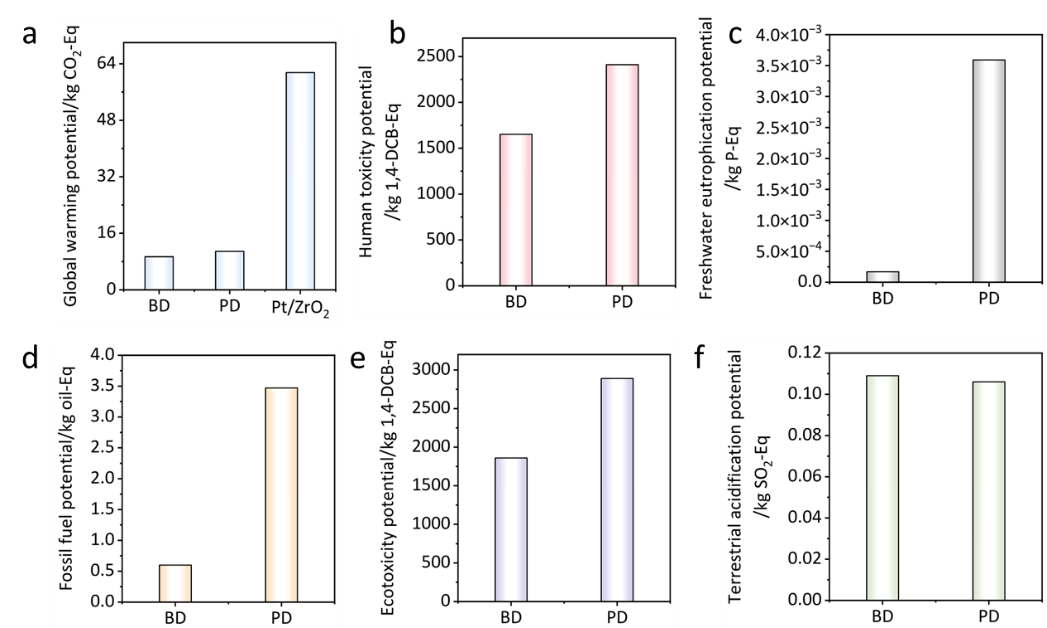


**Fig. S23** (**a-f**) Life cycle assessment indicators for biomass-based FDCA (BD) and petroleum-based terephthalic acid (PD) [S5-S7]


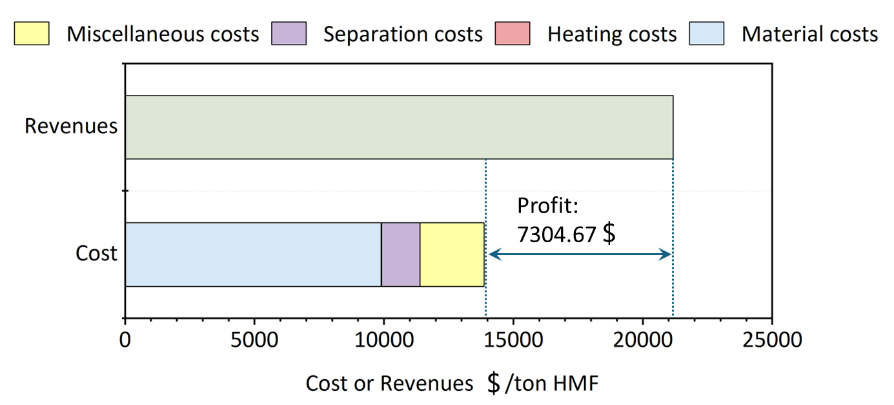


**Fig. S24** TEA analysis


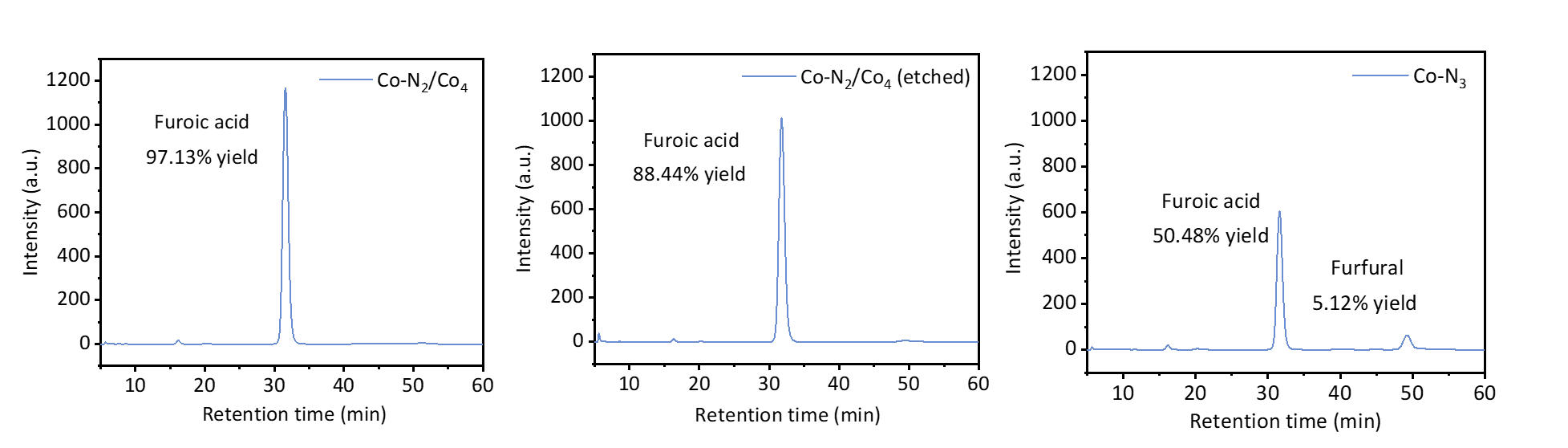

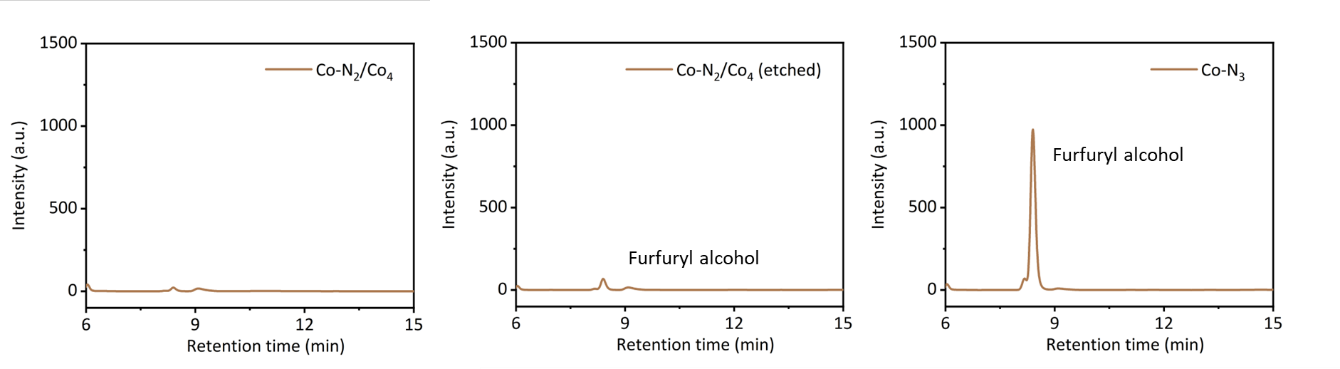


**Fig. S25** HPLC chromatograms of furfuryl alcohol to furoic acid


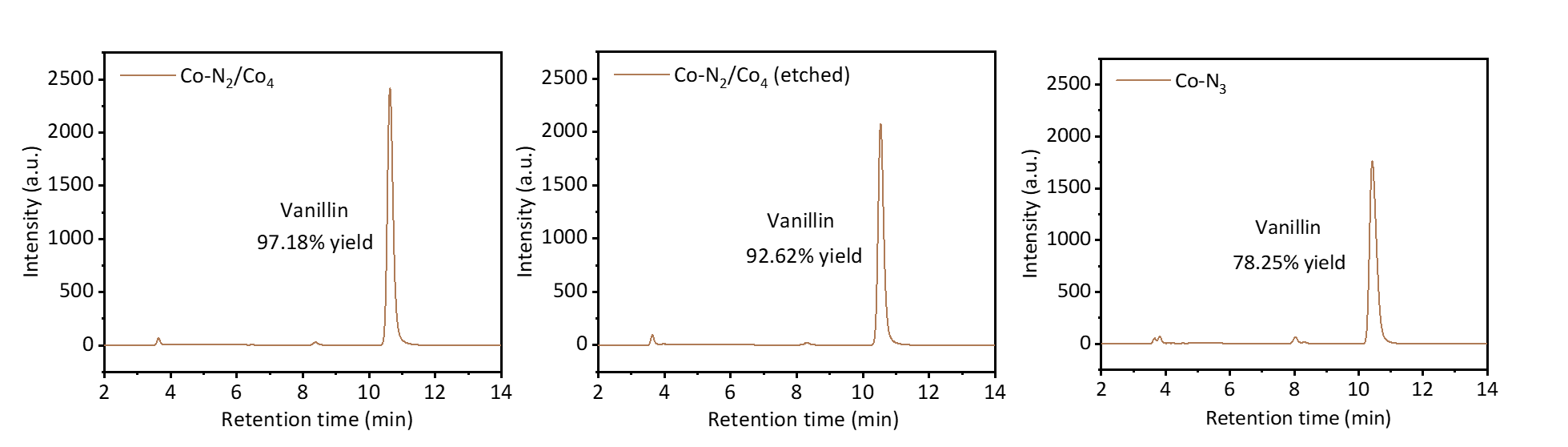


**Fig. S26** HPLC chromatograms of vanillin alcohol to vanillin

**Table S1** Zn and Co contents in the Co catalysts

| Catalysts | Co content | Zn content |
| --- | --- | --- |
| Co-N_4_ SACs | 0.88wt.% | 0.36 wt.% |
| Co-N_3_ SACs | 1.13 wt.% | 0.11 wt.% |
| Co-N_2_/Co_4_ SACs | 1.33 wt.% | / |
| Co-N_2_/Co_6_ SACs | 1.51 wt.% | / |

**Table S2** The atomic proportions of XPS Co and C-N wt.% in the Co catalyst

| Sample | Co-N atomic proportions | Co atomic proportions (wt.%) |
| --- | --- | --- |
| Co-N_4_ | 1.21 | 0.34 |
| Co-N_3_ | 0.46 | 0.16 |
| Co-N_2_/Co_4_ (etched) | 0.32 | 0.13 |

**Table S3** Structural parameters of different samples extracted from the EXAFS fitting

|  | shell | CN | R(Å) | σ^2^(10^-3^Å^2^) | ΔE_0_ (eV) | R factor |
| --- | --- | --- | --- | --- | --- | --- |
| Co-N_3_ | Co-N | 3.2±0.2 | 1.90±0.01 | 4.1±0.4 | -2.1±0.3 | 0.012 |
| Co-N_4_ | Co-N | 3.8±0.4 | 1.92±0.01 | 7.2±1.0 | -4.7±0.4 | 0.014 |

Data ranges: 3.0 ≤ k ≤ 10.0 Å^-1^, 1.0 ≤ R ≤ 2.5 Å. R: bond distance; σ^2^: Debye-Waller factors; R factor: goodness of fit. S_0_^2^ is the amplitude reduction factor (S_0_^2^ =0.75 was obtained by Co foil fitting and applied for the other samples fitting).

**Table S4** Structural parameters of different samples extracted from the EXAFS fitting

|  | S_0_^2^ | Shell | CN* | R(Å) | σ^2^ (10^-3^ Å^2^) | Δ*E*_0_ | R factor |
| --- | --- | --- | --- | --- | --- | --- | --- |
| Co foil | 0.75 | Co-Co | 12 | 2.49±0.01 | 6.3±0.1 | 7.51±0.37 | 0.0019 |
| Co-N_2_/Co_4_ | 0.75 | Co-N | 2.4±0.1 | 1.92±0.01 | 13.5±1.7 | -1.26±0.61 | 0.0021 |
|  |  | Co-Co | 4.1±0.1 | 2.40±0.01 | 13.2±0.6 |  |  |
| Co-N_2_/Co_6_ | 0.75 | Co-N | 1.7±0.1 | 1.93±0.01 | 13.5±4.1 | -5.16±0.94 | 0.0017 |
|  |  | Co-Co | 6.0±0.1 | 2.39±0.01 | 15.4±0.8 |  |  |

*CN*: coordination numbers; *R*: bond distance; *σ*^2^: Debye-Waller factors; Δ*E*_0_: the inner potential correction. R factor: goodness of fit.

**Table S5** The catalytic effect of the Co cluster was verified by poisoning the Co-N_2_ sites with KSCN

| Co_4_ site | HMF conversion | HMFCA yield | FFCA yield | FDCA yield |
| --- | --- | --- | --- | --- |
| 2 mmol (KSCN) | 86.86% | 13.18% | 18.15% | 58.44% |
| 4 mmol (KSCN) | 63.22% | 11.17% | 17.12% | 35.53% |
| 6 mmol (KSCN) | 39.03% | 8.28% | 13.97% | 17.16% |
| Co_6_ site | HMF conversion | HMFCA yield | FFCA yield | FDCA yield |
| 2 mmol (KSCN) | 72.27% | 12.86% | 12.21% | 47.88% |
| 4 mmol (KSCN) | 45.41% | 8.94% | 9.66% | 27.26% |
| 6 mmol (KSCN) | 22.36% | 5.53% | 8.27% | 7.93% |

**Table S6** Catalytic performance of the three catalysts

| Catalysts | Conv. HMF% | Activity (μmol/g/min) |
| --- | --- | --- |
| Co-N_2_/Co_4_ | 8.83 | 117.73 |
| Co-N_2_/Co_4_ (etched) | 5.86 | 78.13 |
| Co-N_2_/Co_4_ (KSCN) | 2.83 | 37.73 |
| Catalysts | Conv. HMFCA% | Activity (μmol/g/min) |
| Co-N_2_/Co_4_ | 9.89 | 131.87 |
| Co-N_2_/Co_4_ (etched) | 1.95 | 26 |
| Co-N_2_/Co_4_ (KSCN) | 5.87 | 78.26 |
| Catalysts | Conv. FFCA% | Activity (μmol/g/min) |
| Co-N_2_/Co_4_ | 7.97 | 102.27 |
| Co-N_2_/Co_4_ (etched) | 6.41 | 85.47 |
| Co-N_2_/Co_4_ (KSCN) | 3.18 | 42.4 |

**Table S7** The reaction results of the Co catalyst at 55 ^o^C, 5bar O_2_ and 5 h

| Samples | HMF conversion | HMFCA yield | FFCA yield | FDCA yield |
| --- | --- | --- | --- | --- |
| Co-N_2_/Co_4_ | 99.87% | 0.95% | 0.16% | 98.76% |
| Co-N_2_/Co_4_ (etched) | 99.58% | 10.62% | 0.21% | 88.42% |
| Co-N_3_ | 99.28% | 13.22% | 1.25% | 86.48% |
| Co-N_4_ | 99.57% | 18.69% | 6.83% | 74.73% |

**Table S8** The reaction results of the Co catalyst at 55 ^o^C and 5 h

| Samples | Oxygen pressure | HMF conversion (%) | HMFCA yield (%) | FFCA yield (%) | FDCA yield (%) |
| --- | --- | --- | --- | --- | --- |
| Co-N_2_/Co_4_ | 1 bar | 99.47 | 10.04 | 15.72 | 74.24 |
|  | 2 bar | 99.68 | 13.76 | 10.55 | 75.66 |
|  | 3 bar | 99.64 | 9.46 | 13.52 | 77.02 |
|  | 4 bar | 99.86 | 10.11 | 0.75 | 89.06 |
|  | 5 bar | 99.87 | 0.95 | 0.16 | 98.76 |
| Co-N_2_/Co_4_ (etched) | 1 bar | 99.02 | 16.97 | 12.94 | 69.09 |
|  | 2 bar | 99.31 | 18.22 | 8.07 | 71.71 |
|  | 3 bar | 99.54 | 11.33 | 15.04 | 74.35 |
|  | 4 bar | 99.62 | 14.19 | 3.65 | 81.78 |
|  | 5 bar | 99.58 | 10.62 | 0.21 | 88.42 |
| Co-N_3_ | 1 bar | 99.08 | 16.58 | 15.72 | 66.80 |
|  | 2 bar | 99.62 | 12.33 | 13.43 | 73.86 |
|  | 3 bar | 99.68 | 10.86 | 10.95 | 77.87 |
|  | 4 bar | 99.73 | 12.42 | 3.58 | 84.00 |
|  | 5 bar | 99.28 | 13.22 | 1.25 | 86.48 |

**Table S9** The Co content in the catalysts after first-run catalysis

| Catalysts | Co content in fresh  catalysts (%) | Co content in regenerated  catalysts (%) |
| --- | --- | --- |
| Co-N_2_/Co_4_ SACs | 1.33 wt.% | 1.25 wt.% |

**Table S10** Statistical data on catalysts for HMF oxidation reactions [S8-S24]

| Catalyst | Reaction conditions | | | HMF conversion (%) | FDCA yield (%) |
| --- | --- | --- | --- | --- | --- |
|  | T(^o^C) | O_2_(bar) | t(h) |  |  |
| *Noble metal-based catalysts* | | | | | |
| Au/TiO_2_ | 80 | 25 | 8 | ＞97 | 87 |
| Au/Ni_x_Co_1_O_y_ | 120 | 5 | 8 | 100 | ＞99 |
| Pd/MnO_2_ | 80 | 1 | 4 | ＞99.9 | 88.1 |
| Au_1_-Pd_1_/ZOC | 80 | 3 | 6 | ＞99.9 | 99.9 |
| Pt_1_-Co@C-N | 100 | 5 | 4 | ＞99 | 99.6 |
| RuO_x_/MnO_x_ | 120 | 10 | 6 | / | 86.56 |
| Pt-Au alloy catalyst | 95 | 10 | 3 | ＞99 | 99 |
| Pt/MnPO | 110 | 10 | 24 | 100 | 98 |
| Ag_1.5_Pd_1.5_/CeO_2_ | 20 | 1 | 4 | 100 | 93 |
| Au/C | 70 | 10 | 4 | ＞99 | ＞85 |
| Pt/γ-Al_2_O_3_ | 100 | 10 | 24 | 100 | 96 |
| Pt/ZrO_2_ | 100 | 4 | 12 | 100 | 97 |
| *Non-noble metal-based catalysts* | | | | | |
| Co/Mn/Br catalysts | 130 | 10 | / | / | 75.8 |
| La-MnO_2_ | 140 | 5 | 4 | 96.3 | 95.4 |
| Co_3_O_4_/Mn_x_Co | 140 | 1 | 24 | ＞99 | 99 |
| a-Co/Cu-NC | 90 | 1 | 8 | ＞99 | 98 |
| CoO_x_-MC | 80 | 5 | 30 | ＞99 | 95.3 |
| (Co_1_→Co_p_)/N-CNTs | 100 | 1 | 8 | 100 | 96 |
| Co-N_2_/Co_4_ SACs | 55 | 5 | 5 | ＞99 | 98.76 |

T=temperature, t=reaction time.

**Table S11** Raw materials required to produce 1kg FDCA

| Raw material | Quantity |
| --- | --- |
| HMF | 1.03 kg |
| Na_2_CO_3_ | 0.1 kg |
| O_2_ | 0.15 kg |
| CoCl_2_·6H_2_O | 6.45×10^-5^kg |
| ZnCl_2_ | 3.69×10^-4^kg |
| Lignin | 0.02 kg |
| NaOH | 0.003 kg |
| Melamine | 0.18 kg |
| Electricity | 21.8 kWh |

**Table S12** Furfuryl alcohol and vanillin alcohol are oxidized to furoic acid and vanillin

|  | Furoic acid yield (%) | | Furfural yield (%) | | Furfuryl alcohol conversion (%) |
| --- | --- | --- | --- | --- | --- |
| Co-N_2_/Co_4_ | 97.13 | | 0.49 | | 99.62 |
| Co-N_2_/Co_4_ (etched) | 88.44 | | 0.57 | | 91.01 |
| Co-N_3_ | 50.48 | | 5.12 | | 57.60 |
|  | | Vanillin alcohol conversion (%) | | Vanillin yield (%) | |
| Co-N_2_/Co_4_ | | ＞99 | | 97.18 | |
| Co-N_2_/Co_4_ (etched) | | ＞98 | | 92.62 | |
| Co-N_3_ | | ＞99 | | 78.25 | |

**Supplementary Note S1**

A simplified techno-economic analysis (TEA) was conducted to evaluate the feasibility of the HMF oxidation process.

1. Material costs

Material costs: 1 ton HMF (9850 $/t), 9 ton water (0.7 $/t) and 0.11 ton Na_2_CO_3_ (300 $/t). Therefore, the capital costs component can be calculated as:

Material cost = 9889.3 $

2. Heating costs:

The heating cost mainly consists of heating energy consumption and heat loss energy consumption.

Heating energy consumption formula:

Q_heating_ = m × c × ΔT=9×10^6^×4.184×95=3.58×10^6^kJ

Specific heat capacity of water: c = 4.184 J/g·°C (standard value).

Energy Consumption for Heat Loss to Maintain Temperature:

Formula:

Q_loss_=P_loss_ × t=(U × ΔT) × t=0.03×95×43200=123.12kJ

Typical range of the heat loss coefficient U for insulation of a reactor: U≈0.02~0.05 W/°C. Here, an intermediate value is taken: U=0.03 W/°C

Q_total_=Q_heating_+Q_loss_≈3.58×10^6^kJ

The energy input of 3.58×10⁶ kJ required for the reactor operation corresponds to a coal cost of approximately 18.3 $.

Heating costs=18.3 $

3. Separation costs:

The separation process, comprising acidification-induced precipitation, solid-liquid separation, washing, and drying, generates dominant costs from material consumption, energy input, and wastewater pretreatment. These combined operational expenditures account for approximately 15% of the raw material cost.

Separation costs=1483.4 $

4. Miscellaneous costs:

In the HMF oxidation process, miscellaneous costs including capital costs, maintenance costs and operating costs account for approximately 25% of the raw material costs

Miscellaneous cost = 2472.33 $

5. Total plant gate levelized cost:

Finally, the total cost can now be calculated by adding up all 4 components:

Toal cost = 9889.3 $ + 18.3 $ + 1483.4 $ +2472.33 $ = 13863.33 $

**Product value:**

After catalysis, 1 ton of HMF feedstock finally gave 1.08 ton FDCA (19600 $/t).

Product value = 21168 $

**Potential profit:**

Therefore, the total income of catalysis per ton of HMF can be calculated as follows:

Toal profit = Product value – Toal cost = 21168 – 13863.33 = 7304.67 $

**Supplementary References**

1. G. Kresse, J. Hafner, *Ab initio*molecular dynamics for liquid metals. Phys. Rev. B **47**(1), 558–561 (1993). <https://doi.org/10.1103/physrevb.47.558>
2. G. Kresse, J. Hafner, *Ab initio* molecular-dynamics simulation of the liquid-metal-amorphous-semiconductor transition in germanium. Phys. Rev. B **49**(20), 14251–14269 (1994). <https://doi.org/10.1103/physrevb.49.14251>
3. J.P. Perdew, K. Burke, M. Ernzerhof, Generalized gradient approximation made simple. Phys. Rev. Lett. **77**(18), 3865–3868 (1996). <https://doi.org/10.1103/physrevlett.77.3865>
4. S. Grimme, J. Antony, S. Ehrlich, H. Krieg, A consistent and accurate *ab initio* parametrization of density functional dispersion correction (DFT-D) for the 94 elements H-Pu. J. Chem. Phys. **132**(15), 154104 (2010). <https://doi.org/10.1063/1.3382344>
5. M.G. Davidson, S. Elgie, S. Parsons, T.J. Young, Production of HMF, FDCA and their derived products: a review of life cycle assessment (LCA) and techno-economic analysis (TEA) studies. Green Chem. **23**(9), 3154–3171 (2021). <https://doi.org/10.1039/d1gc00721a>
6. K. Wiranarongkorn, K. Im-Orb, D. Saebea, Y. Patcharavorachot, A. Arpornwichanop, Energy analysis and life cycle assessment of furfural and 5-hydroxymethylfurfural integrated biorefinery processes with heat pump-assisted reactive distillation. Energy **319**, 134720 (2025). <https://doi.org/10.1016/j.energy.2025.134720>
7. Y. Lei, G. Zhu, X. Liu, X. Wu, B. Fu et al., Comparative assessment of 5-hydroxymethylfurfural Production: Economic viability and environmental performance of five conceptual bioprocesses. Biomass Bioenergy **201**, 108118 (2025). <https://doi.org/10.1016/j.biombioe.2025.108118>
8. H. Ban, Y. Zhang, S. Chen, Y. Cheng, T. Pan et al., Production of 2, 5-furandicarboxylic acid by optimization of oxidation of 5-methyl furfural over homogeneous Co/Mn/Br catalysts. ACS Sustainable Chem. Eng. **8**(21), 8011–8023 (2020). <https://doi.org/10.1021/acssuschemeng.0c02574>
9. H. Chen, J. Shen, K. Chen, Y. Qin, X. Lu et al., Atomic layer deposition of Pt nanoparticles on low surface area zirconium oxide for the efficient base-free oxidation of 5-hydroxymethylfurfural to 2, 5-furandicarboxylic acid. Appl. Catal. A Gen. **555**, 98–107 (2018). <https://doi.org/10.1016/j.apcata.2018.01.023>
10. T. Gao, Y. Yin, G. Zhu, Q. Cao, W. Fang, Co_3_O_4_ NPs decorated Mn-Co-O solid solution as highly selective catalyst for aerobic base-free oxidation of 5-HMF to 2, 5-FDCA in water. Catal. Today **355**, 252–262 (2020). <https://doi.org/10.1016/j.cattod.2019.03.065>
11. Z. Gui, W. Cao, S. Saravanamurugan, A. Riisager, L. Chen et al., Efficient aerobic oxidation of 5-hydroxymethylfurfural in aqueous media with Au–Pd supported on zinc hydroxycarbonate. ChemCatChem **8**(23), 3636–3643 (2016). <https://doi.org/10.1002/cctc.201600852>
12. Q. Hou, T. Gao, H. Zhang, Q. Tang, W. Fang, Pt nanoparticles in cooperation with Mn-P composite drive base-free selective oxidation of 5-hydroxymethylfurfural. Catal. Today **443**, 114990 (2025). <https://doi.org/10.1016/j.cattod.2024.114990>
13. Y. Jin, S. Sarina, H. Liu, W. Martens, E.R. Waclawik et al., Aerobic oxidation of 5-hydroxymethyl-furfural to 2, 5-furandicarboxylic acid at 20 °C by optimizing adsorption on AgPd alloy nanoparticle catalysts. ACS Catal. **12**(18), 11226–11238 (2022). <https://doi.org/10.1021/acscatal.2c03457>
14. T. Jing, S. Yang, T. Li, Y. Wan, H. Jia et al., Atomic Co/Cu dual-metal for rapid conversion of 5-hydroxymethylfurfural under ambient pressure. Adv. Funct. Mater. **34**(44), 2407335 (2024). <https://doi.org/10.1002/adfm.202407335>
15. X. Liao, J. Hou, Y. Wang, H. Zhang, Y. Sun et al., An active, selective, and stable manganese oxide-supported atomic Pd catalyst for aerobic oxidation of 5-hydroxymethylfurfural. Green Chem. **21**(15), 4194–4203 (2019). <https://doi.org/10.1039/C9GC01674K>
16. X. Liu, M. Zhang, Z. Li, CoOx-MC (MC = mesoporous carbon) for highly efficient oxidation of 5-hydroxymethylfurfural (5-HMF) to 2, 5-furandicarboxylic acid (FDCA). ACS Sustainable Chem. Eng. **8**(12), 4801–4808 (2020). <https://doi.org/10.1021/acssuschemeng.9b07443>
17. C. Megías-Sayago, A. Lolli, D. Bonincontro, A. Penkova, S. Albonetti et al., Effect of gold particles size over Au/C catalyst selectivity in HMF oxidation reaction. ChemCatChem **12**(4), 1177–1183 (2020). <https://doi.org/10.1002/cctc.201901742>
18. P. Rapado, C. Lois, M. Cano, L. Faba, S. Ordóñez, Biomass to plastics: From HMF to fructose for the synthesis of 2, 5-furandicarboxylic acid over Au/TiO_2_. Catal. Today **445**, 115036 (2025). <https://doi.org/10.1016/j.cattod.2024.115036>
19. R. Sahu, P.L. Dhepe, Synthesis of 2, 5-furandicarboxylic acid by the aerobic oxidation of 5-hydroxymethyl furfural over supported metal catalysts. React. Kinet. Mech. Catal. **112**(1), 173–187 (2014). <https://doi.org/10.1007/s11144-014-0689-z>
20. S. Yang, C. Wu, J. Wang, H. Shen, K. Zhu et al., Metal single-atom and nanoparticle double-active-site relay catalysts: design, preparation, and application to the oxidation of 5-hydroxymethylfurfural. ACS Catal. **12**(2), 971–981 (2022). https://doi.org/10.1021/acscatal.1c05236
21. L. Yu, H. Chen, Z. Wen, M. Jin, X. Ma et al., Efficient aerobic oxidation of 5-hydroxymethylfurfural to 2, 5-furandicarboxylic acid over a nanofiber globule La-MnO_2_ catalyst. Ind. Eng. Chem. Res. **60**(4), 1624–1632 (2021). <https://doi.org/10.1021/acs.iecr.0c05561>
22. H. Zhang, Y. Wang, Q. Zhang, B. Gu, Q. Tang et al., Synergy in magnetic Ni_x_Co_1_O_y_ oxides enables base-free selective oxidation of 5-hydroxymethylfurfural on loaded Au nanoparticles. J. Energy Chem. **78**, 526–536 (2023). <https://doi.org/10.1016/j.jechem.2022.11.057>
23. X. Zhong, P. Yuan, Y. Wei, D. Liu, D. Losic et al., Coupling natural halloysite nanotubes and bimetallic Pt–Au alloy nanoparticles for highly efficient and selective oxidation of 5-hydroxymethylfurfural to 2, 5-furandicarboxylic acid. ACS Appl. Mater. Interfaces **14**(3), 3949–3960 (2022). <https://doi.org/10.1021/acsami.1c18788>
24. W. Zhu, Y. Meng, C. Yang, J. Zhao, H. Wang et al., Effect of coordination environment surrounding a single Pt site on the liquid-phase aerobic oxidation of 5-hydroxymethylfurfural. ACS Appl. Mater. Interfaces **13**(41), 48582–48594 (2021). <https://doi.org/10.1021/acsami.1c12329>
